# Interleukin-37 promotes colitis-associated carcinogenesis via SIGIRR-mediated cytotoxic T cells dysfunction

**DOI:** 10.1038/s41392-021-00820-z

**Published:** 2022-01-20

**Authors:** Zhen Wang, Fan-lian Zeng, Ya-wen Hu, Xiao-yan Wang, Fu-lei Zhao, Pei Zhou, Jing Hu, Yuan-yuan Xiao, Zhong-lan Hu, Ming-feng Guo, Xiao-qiong Wei, Xiao Liu, Nong-yu Huang, Jun Zhang, Shu-wen Chen, Juan Cheng, Hua-ping Zheng, Hong Zhou, Qi-xiang Zhao, Chen Zhang, Yan Hao, Song Zou, Yi-yue Gui, Jia-dong Yu, Lin-na Gu, Cheng-cheng Yue, Hao-zhou Zhang, Wen-ling Wu, Yi-fan Zhou, Xi-kun Zhou, Guo-bo Shen, Xiu Teng, Jiong Li

**Affiliations:** 1 State Key Laboratory of Biotherapy and Cancer Center, West China Hospital, West China Medical School, Sichuan University and Collaborative Innovation Center for Biotherapy, Chengdu, Sichuan 610041 China; 2 Department of Liver Surgery & Liver Transplantation, West China Hospital, Sichuan University and Collaborative Innovation Center of Biotherapy, Chengdu, Sichuan 610041 China; 3 Laboratory of Liver Surgery, West China Hospital, Sichuan University, Chengdu, Sichuan 610041 China; 4 Department of Obstetrics and Gynecology, West China Second University Hospital, Sichuan University, Chengdu, 610041 China; 5 Key Laboratory of Birth Defects and Related Diseases of Women and Children, Ministry of Education, Sichuan University, Chengdu, 610041 China; 6 Department of Cardiovascular Medicine, West China Hospital, Sichuan University, Chengdu, Sichuan 610041 China; 7 Laboratory of Human Disease and Immunotherapies, West China Hospital, Sichuan University, Chengdu, Sichuan 610041 China

**Keywords:** Tumour immunology, Adaptive immunity, Cancer microenvironment

## Abstract

Interleukin-37b (hereafter called IL-37) was identified as fundamental inhibitor of natural and acquired immunity. The molecular mechanism and function of IL-37 in colorectal cancer (CRC) has been elusive. Here, we found that IL-37 transgenic (IL-37tg) mice were highly susceptible to colitis-associated colorectal cancer (CAC) and suffered from dramatically increased tumor burdens in colon. Nevertheless, IL-37 is dispensable for intestinal mutagenesis, and CRC cell proliferation, apoptosis, and migration. Notably, IL-37 dampened protective cytotoxic T cell-mediated immunity in CAC and B16-OVA models. CD8^+^ T cell dysfunction is defined by reduced retention and activation as well as failure to proliferate and produce cytotoxic cytokines in IL-37tg mice, enabling tumor evasion of immune surveillance. The dysfunction led by IL-37 antagonizes IL-18–induced proliferation and effector function of CD8^+^ T cells, which was dependent on SIGIRR (single immunoglobulin interleukin-1 receptor-related protein). Finally, we observed that IL-37 levels were significantly increased in CRC patients, and positively correlated with serum CRC biomarker CEA levels, but negatively correlated with the CD8^+^ T cell infiltration in CRC patients. Our findings highlight the role of IL-37 in harnessing antitumor immunity by inactivation of cytotoxic T cells and establish a new defined inhibitory factor IL-37/SIGIRR in cancer-immunity cycle as therapeutic targets in CRC.

## Introduction

Colorectal cancer is a main cause of morbidity and mortality worldwide.^1^ CD8^+^ cytotoxic T lymphocytes (CTLs) are preferred immune cells for attacking cancer. Several studies have identified CD8^+^ CTLs density in the tumor and in the invasive margin for assessing recurrence risk of CRC.^2^ CD8^+^ CTL infiltration can be as an independent predictor of favorable survival outcomes in patients with CRC.^3^ Moreover, hot tumors with the high degree of CD8^+^ CTL infiltration displaying high response rates to the immune checkpoint blockers.^4^ These findings imply that CD8^+^ CTLs are key defender of CRC. CD8^+^ CTLs kills tumor cells primarily through IFN-γ-mediated or perforin-mediated mechanisms.^5^ The IFN-γ, as a CD8^+^ CTL–derived cytotoxic cytokine, can initiate apoptosis in tumor cells by activating JAK-STAT1-caspase3 cascade.^6^ In addition, the perforin-mediated tumor killing mechanisms is achieved through degranulation of CD8^+^ CTLs, such as releasing degranulated cargos, which including the cytotoxic proteins perforin and granzymes. Perforin promotes the formation of transmembrane pores, leading to granzymes enter tumor cells and initiate apoptosis.^7^ The degranulation capacity of CD8^+^ CTLs was assessed by cell surface expression of CD107.^8^ The dysfunction of CD8^+^ CTLs constitutes an important factor for tumor escape.

Cytokines, as secreted proteins, can provide critical clues to immune cells, therefore they are attractive target candidates for cancer immunotherapy. IL-18, which is a member of the IL-1 family, drives MyD88–IRAK4–JNK signaling through heterodimer receptor comprised of IL-18Rα and IL-18Rβ subunits. Previous studies have reported that IL-18, in synergy with IL-12, stimulates cytotoxic cytokine IFN-γ production in CD8^+^ T cells.^9^ It has previously been observed that recombinant IL-18 inhibit tumor progression in pre-clinical models of CRC.^10^ Moreover, recombinant IL-18 has good safety and well tolerability as a cancer drugs in clinical trials.^11^ Recently, an engineered decoy-resistant IL-18 exhibits potential anticancer effects in vivo, which highlight the significance of IL-18 pathway for therapeutic immune interventions of tumor.^12^ These findings have redefined the crucial role of IL-18 as an antitumor cytokine that fundamentally alters the immune tumor microenvironment.

Interleukin-37 (IL-37) is a new IL-1 family member,^13,14^ as a recently identified IL-18 inhibit factor, disturbs the expression of IL-18-dependent inflammatory factors (TNF-α, IL-1β, and IL-6) in renal tubular epithelial cells.^15^ Previously research proved that the tripartite complex composed of IL-37, SIGIRR, and IL-18Ra is indispensable for the function of IL-37.^16^ SIGIRR is an orphan receptor, overexpression of SIGIRR inhibits IL-18 signaling in Jurkat and HepG2 cells.^17^ These findings imply that SIGIRR might play a pivotal function for IL-37 to inhibit IL-18 signaling. IL-37 monitored natural immunity, as well as emerges as a key regulatory molecule of adaptive immunity. Feng et al. indicated that IL-37 diminished Th1 response but promoted the Th2 cytokines (IL-13 and IL-4) production in ConA induce liver injury.^18^ IL-37 stimulation elevated the Tregs cell proportion in subjects with infectious disease and hypersensitivity.^19,20^ These findings suggested IL-37 function as important regulatory factor on T cell immune response. Considering the anti-inflammatory properties of IL-37, it has been found that IL-37 can influence the development of some cancers, such as non-small cell lung cancer, hepatocellular carcinoma, cervical cancer, and colon cancer.^21,22^ However, immunological roles and functional mechanism of IL-37 have remained elusive in tumor microenvironment. Specifically, the role of IL-37 for CD8^+^ CTL tumor immunosurveillance is not well understood.

Discovering and understanding key molecules and biological processes will provide invaluable guidance for the development of effective therapies in cancer. Many IL-1 family members have shown the crucial regulatory roles in antitumor immune responses, thereby effecting tumor immune escape. The functional mechanism of the novel IL-1 family member IL-37 in the tumor microenvironment is still unclear. Here, we confirmed that anti-inflammatory cytokine IL-37 can function as a critical inducer of dysfunctional cytotoxic CD8^+^ T cells, thus promoted colitis-associated carcinogenesis. Furthermore, this study revealed that IL-37 antagonized IL-18-induced cytotoxic activity of CD8^+^ T cells via its inhibitory receptor SIGIRR. These findings enhance knowledge regarding IL-1 family in tumor microenvironment, highlighting the role of IL-37 in harnessing antitumor immunity. Moreover, the new defined inhibitory factor IL-37/SIGIRR in the cancer-immunity cycle is established as therapeutic targets in colorectal cancer.

## Results

### The colon epithelium of IL-37-transgenic mice exhibits balanced homeostasis

**Fig. 1:**
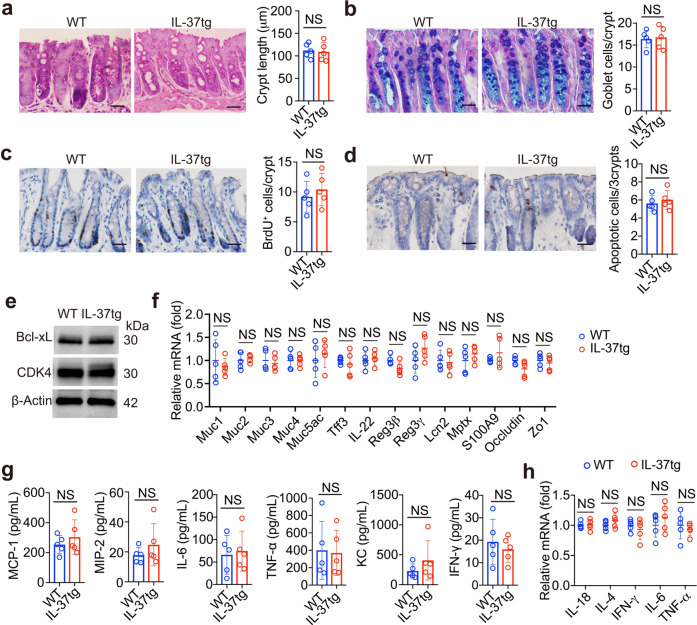
The colon epithelium of IL-37-transgenic mice exhibits balanced homeostasis. **a** Representative H&E-stained sections (left, scale bar: 50 μm) of the distal colon of IL-37tg mice and WT mice, and quantification of the crypt length (right) is shown. At least 15 well-oriented crypts were measured on slides from each mouse, *n* = 5/group. **b** Representative alcian blue and PAS staining of distal colon sections obtained from IL-37tg mice and WT mice (left, scale bar: 50 μm). Statistical analysis of enumeration of goblet cells per crypt (right), at least 15 well-oriented crypts were measured on slides from each mouse, *n* = 5/group. **c** 50 mg/kg BrdU was administrated to IL-37tg mice and WT mice by intraperitoneal injection. The colon sections were harvested and stained for BrdU-positive cells at 24 h after BrdU injection. Representative BrdU-stained sections are shown (left, scale bar: 50 μm). Statistical analysis of BrdU-positive cells per crypt (right), at least 15 well-oriented crypts were counted on slides from each mouse, *n* = 5/group. **d** DNA fragmentation in the colon of IL-37tg mice and WT mice was detected by in situ TUNEL assay. Apoptotic cells were stained brown and nuclei were stained blue by hemotoxylin, at least 15 Well-oriented crypts were counted on slides from each mouse, scale bar: 50 μm, n = 5/group. **e** Immunoblotting was performed to detect CDK4 and Bcl-xL in colon epithelial cells from IL-37tg and WT mice. β-Actin was used as a loading control. **f** qRT-PCR analysis of indicated genes from the colons of IL-37tg mice and WT mice, *n* = 5/group. **g** The same amount of colon tissue from IL-37tg mice and WT mice was cut into small pieces and incubated in serum-free RPMI medium for 24 h. Secreted chemokines and cytokines in the medium were measured by ELISA assay, *n* = 5/group. **h** qRT-PCR analysis was performed for each indicated gene in colon tissues of IL-37tg mice and WT mice. *n* = 5/group. All data are presented as mean ± SD. Statistics analyzed by Two-tailed Student’s *T*-test. NS, not significant

To identify the role of IL-37 in colon homeostasis, IL-37 transgenic (IL-37tg) mice was generated and identified (Supplementary Fig. S1a–c). Compared with WT mice, IL-37tg mice show normal growth and development (Supplementary Fig. S1d, e), and similar immune cell distribution (Supplementary Fig. S1f). IL-37 expression profiling was identified in IL-37tg mice at rest state, showing high IL-37 expression in colon tissue, spleen tissue, and PBMC (Supplementary Fig. S1g, h). IL-37 gene copies were found in the colon of IL-37tg mice (Supplementary Fig. S1i), and immunohistochemical results show that IL-37 was primary expression in intestinal epithelial cells, many inflammatory cells show positive staining (Supplementary Fig. S1j). No difference was observed in the length of crypts (Fig. 1a) and the number of goblet cells (Fig. 1b) between IL-37tg and WT mice. In addition, qPCR results showed that IL-37tg and WT colon tissues had similar expression levels of the stem cell marker (Lgr5) and the Paneth cell markers (CD24 and c-Kit) (Supplementary Fig. S1k). Moreover, the proliferating epithelial cells (BrdU^+^) in crypts exhibit similar amount and located at the bottom of the crypts in both strains (Fig. 1c). Compared to WT colon epithelium, IL-37tg colon epithelium had similar number of apoptotic cells at upper crypts (Fig. 1d). Furthermore, western blotting results showed that IL-37tg colon epithelial cells had similar expression of anti-apoptotic protein Bcl-xL and cell cycle protein CDK4 compared to that in WT cells (Fig. 1e). These results indicate that colon epithelium of IL-37tg do not display dysregulation of proliferation and apoptosis at basal state.

No significant differences were found in mRNA levels of mucins (Muc1, Muc2, Muc3, Muc4, and Muc5ac), goblet cell associated gene Tff3, epithelium-healing genes (IL-22, Reg3β, Reg3γ, Lcn2, Mptx, and S100A9), and epithelial tight junction proteins (occludin and ZO1) between WT and IL-37tg mice at rest time (Fig. 1f), implying no defects in the barrier function in IL-37tg mice.

We further investigated whether IL-37 affects inflammation in the colonic mucosa homeostasis. Compared to WT colon, IL-37tg colonic mucosa showed did not significantly change release levels of chemokines (MCP-1, MIP-2, and KC) and cytokines (IL-6, TNF-a, and IFN-γ) (Fig. 1g). Similarly, there was no significant difference in expression levels of the IL-18, IL-4, IFN-γ, IL-6, TNF-a (Fig. 1h). Taken together, IL-37 transgene does not influence epithelial homeostasis and physiological immune system defenses in the colonic mucosa at basal state.

### IL-37 renders mice more susceptible to AOM/DSS-induced colorectal cancer

**Fig. 2:**
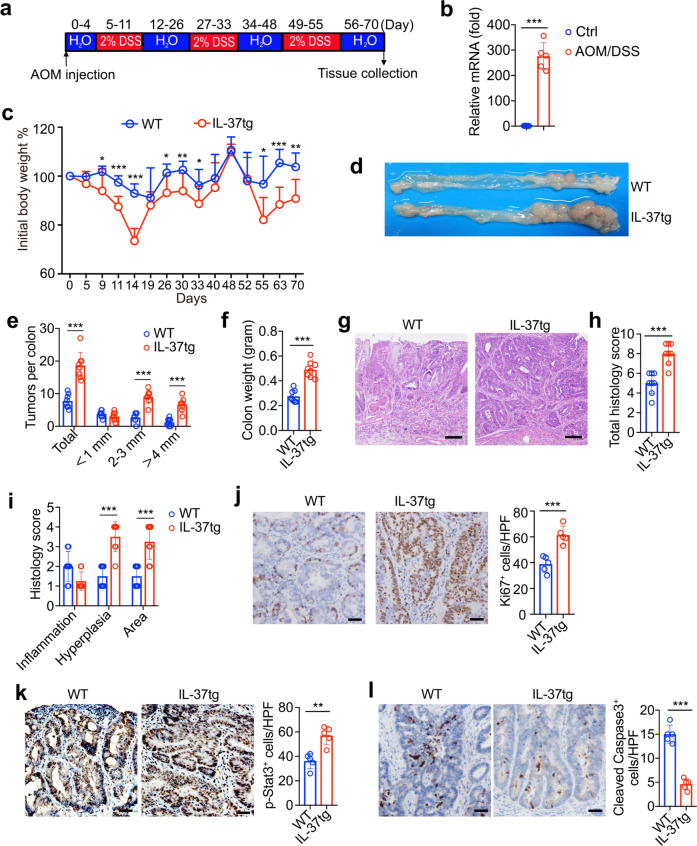
Enhanced tumorigenesis in IL-37tg mice. **a** Details of the azoxymethane/dextran sodium sulfate (AOM/DSS) treatment used for the induction of inflammation-associated colorectal cancer. IL37tg mice and WT mice were injected with AOM on day 0, and were then given a 2% DSS solution during three 6-day cycles as described in experimental procedures. **b** qRT-PCR analysis of IL-37 expression in the colon of IL-37tg mice after treatment with or without AOM/DSS, *n* = 5/group. **c** The body weight loss of IL-37tg mice and WT mice following injected with AOM on day 0 and administered 3 rounds of 2% DSS in drinking water, *n* = 8/group. **d** Representative image of the distal colon at day 70 after AOM/DSS administration, tumor development in the colon was determined. **e** Quantification of the number of tumors in the colon at day 70 after AOM/DSS administration, *n* = 8/group. **f** Colon weight was determined in IL-37tg mice and WT mice at day 70 after AOM/DSS administration, *n* = 8/group. **g** Colon tissue sections of IL-37tg mice and WT mice by H&E staining at day 70 after AOM/DSS administration. Scale bar, 100 μm. **h**, **i** Total histological scores (**h**) and scores for different parameters (**i**) of IL-37tg mice and WT mice at day 70 after AOM/DSS administration, *n* = 8/group. **j**–**l** Representative Ki67 (**j**), p-Stat3 (**k**), Cleaved Caspase3 (**l**) immunohistochemistry of colon in IL-37tg mice and WT mice at day 70 after AOM/DSS administration, scale bars: 100 μm. Positive cells were quantified by counting the stained dots, *n* = 8/group. All data are presented as mean ± SD. Statistics analyzed by Two-tailed Student’s *T*-test. *P < 0.05; **P < 0.01; ***P < 0.001

As McNamee et al reported hIL-37tg mice were protected from DSS-induced colitis,^23^ it is widely accepted that chronic inflammation is closely involved in tumorigenesis. To identify the contribution of IL-37 to CRC, AOM/DSS-induced colitis-associated colorectal cancer (CAC) model was used (Fig. 2a), IL-37 expression was increased in colon of IL-37tg mice after treatment AOM/DSS (Fig. 2b). IL-37tg mice had more severe weight loss during each DSS round compare to WT mice (Fig. 2c). Tumor burden was significantly increased in IL-37tg mice compared with those in WT mice (Fig. 2d). In addition to bearing more tumors, IL-37tg mice had larger tumors, for the number of tumors which greater than 2 mm in diameter were more than that in WT mice (Fig. 2e), we also found an increase in colon weight in IL-37tg mice (Fig. 2f).

Histological analysis indicated that IL-37tg mice showed more serious pathological damage after tumorigenesis (Fig. 2g–i). IL-37tg stimulated cellular proliferation as demonstrated by the increased number of cells that are proliferation marker Ki67 positive (Fig. 2j). As previously reported, activated Stat3 promoted cell proliferation and increased growth and invasive capacity of tumors.^24^ We observed that the p-Stat3-positive cells were higher in tumors of IL-37tg mice compared with that in WT controls (Fig. 2k). Analysis of cleaved Caspase-3 staining revealed that apoptotic cells were decreased in tumors of IL-37tg mice (Fig. 2l). Altogether, these results suggested a pivotal role for IL-37 in promoting tumor progression in the CAC.

To identify the role of IL-37 in colitis phase. DSS-induced colitis mouse model was used, results show that the expression level of IL-37 was significantly higher in the colon after treatment with DSS than in the steady state (Supplementary Fig. S2a). IL-37tg mice were extremely susceptible to DSS treatment, the body weight of IL-37tg mice had lost more than WT mice (Supplementary Fig. S2b), IL-37tg mice showed severe signs of colitis, including diarrhea, rectal bleeding and drastically shortened colon (Supplementary Fig. S2c–e). These results demonstrated that exposure of DSS elicited more severe colitis symptom in IL-37tg mice.

We found that colonic immune cells infiltrates was decreased in IL-37tg mice compared to those in WT mice (Supplementary Fig. S2f), which was consistent with reduced proinflammatory cytokines secretion in colons of IL-37tg mice (Supplementary Fig. S2g). These results implied that IL-37, as a fundamental inhibitor of innate immunity, impaired the colonic immune response. Notably, we found that IL-37tg mice displayed more severe colon injury (Supplementary Fig. S2h), and showed a defect in intestinal epithelial restitution by claudin-3 staining in DSS model (Supplementary Fig. S2i). Consistently, intestinal epithelial cell proliferation was impaired in IL-37tg mice by Ki67 staining (Supplementary Fig. S2j). To further confirm these histological observations, we detected epithelial tissue disruption and permeability by the FITC-dextran measurement, showing that plasma FITC fluorescence was significant increased in IL-37tg mice compared to WT mice (Supplementary Fig. S2k). In conclusion, IL-37 is disadvantage to maintain colonic tissue repair in IL-37tg mice after treated with DSS.

### IL-37 inhibited the activation of tumor-protective CD8^+^ T cells in colon and mesenteric lymph nodes (MLN)

The initiation of carcinogen AOM-induced tumorigenesis was assessed by quantifying γH2AX labeling.^25^ As shown in Supplementary Fig. S3a and S3b, the percentage of γH2AX^+^ cells were similar in colons of IL-37tg mice and WT mice. Previous research has indicated that levels of DNA mismatch repair gene expression in epithelial cells after DNA damage is associated with frequency of oncogenic mutations induced by AOM.^26^ We found a similar mismatch DNA repair genes expression in tumors of IL-37tg mice and WT mice, including poly ADP-ribose polymerase (Parp) family members (Parp1, msh2) and mismatch repair (MMR) proteins (Msh3, Mlh1), as well as genes involved in double-strand DNA repair (ataxia telangiectasia mutated, Atm; ataxia telangiectasia and Rad3 related, ATR) (Supplementary Fig. S3c). These data implied that tumor outgrowth in IL-37tg mice was irrelevant to higher colonotropic mutagenicity or increased DNA damage.

We further investigated whether IL-37 had a positive effect on the overproliferation of colon tumor cells. No obvious change in proliferation rate was observed through CCK8 in IL-37 stable expressing human LoVo colon carcinoma cells (LV-IL-37/LoVo) and control cells (LV-Ctrl/LoVo) (Supplementary Fig. S4a). Flow cytometry analysis of the percentage of G1, S and G2 cells, and percentage of apoptotic cells, suggested that IL-37 did not disturb the cell cycle and apoptosis in Lovo cells (Supplementary Fig. S4b, c). Moreover, the wound healing assay showed similar migratory ability of LV-IL-37/LoVo cells and LV-Ctrl/LoVo cells (Supplementary Fig. S4d). Injecting LV-IL-37/LoVo cells and LV-Ctrl/LoVo cells subcutaneously into nude mice. Overexpression of IL-37 failed to change tumor volumes and weights compared to those in controls. (Supplementary Fig. S4e, f). In addition, LV-IL-37/LoVo nude mice model exhibited similar ki67 expression compared to that in the LV-Ctrl/LoVo group (Supplementary Fig. S4g). LV-IL-37/LoVo cells and LV-Ctrl/LoVo cells have similar c-myc as well as p-p65, pSmad3L and pSmad3C level (Supplementary Fig. S4h, I), although IL-37 inhibited liver tumor growth via converting pSmad3 isoform pathway from pSmad3L tumor-promoting signal to pSmad3C tumor-suppressing signal.^27^ These results can be drawn that overexpression of IL-37 has negligible effects on proliferation, apoptosis and motility of colon carcinoma cells at rest state.

**Fig. 3:**
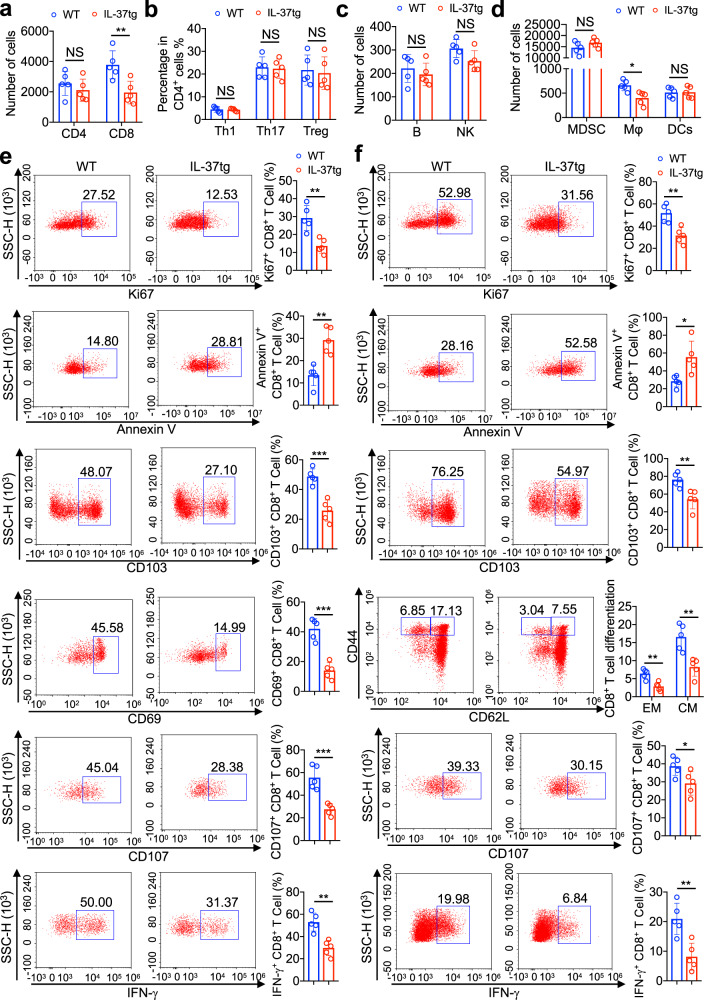
IL-37 inhibited the activation of CD8^+^ cytotoxic T cells in AOM/DSS-induced CRC mouse model. **a** IL-37tg mice and WT mice were treated with AOM/DSS for 70 days, absolute cell numbers of CD4^+^ T cells and CD8^+^ T cells in the tumor-bearing colon tissue as assessed by flow cytometry. A total of 200 000 live colonic cells were acquired to normalise the baselines for all samples, *n* = 5/group. **b** Quantification of intracellular FACS analysis of CD4^+^ T cells (Th1), IL-17A-producing CD4^+^ T cells (Th17), and Foxp3^+^ CD4^+^ T cells (Treg) in the colon of IL-37tg mice and WT mice which were treated with AOM/DSS for 70 days, *n* = 5/group. **c**, **d** IL-37tg mice and WT mice were treated with AOM/DSS for 70 days, absolute cell numbers of CD19^+^ cells (B cells) and NK1.1^+^ cells (NK cells), CD11B^+^Gr-1^+^ (MDSCs), CD11B^+^F4/80^+^ cell (Mφ cells) and CD11C^+^MHC-II^+^ cell (DCs) in the colon as assessed by flow cytometry. A total of 200,000 live colonic cells were acquired to normalise the baselines for all samples, *n* = 5/group. **e**, **f** IL-37tg mice and WT mice were treated with AOM/DSS for 70 days, representative and quantification of FACS analysis of CD8^+^ T cells expressing proliferation marker Ki67, apoptosis marker Annexin V, integrin associated with intestinal T cell retention CD103, activation marker (CD44, CD62L or CD69), and effector molecules (CD107 and IFN-γ) in the tumor-bearing colon tissue (**e**) and mesenteric lymph nodes (**f**), *n* = 5/group. All data are presented as mean ± SD. Statistics analyzed by Two-tailed Student’s *T*-test. *P < 0.05; **P < 0.01; ***P < 0.001. NS, not significant

To understand immune mechanisms of pro-tumor effect of IL-37, various immune cells in tumors of WT and IL-37tg mice were analyzed by flow cytometry (Supplementary Fig. S5). The significantly lower number of CD8^+^ T cells was found within colon tissue of AOM/DSS-treated IL-37tg mice than AOM/DSS-treated WT mice, but no significant changes were found in CD4^+^ T cells (Fig. 3a). And no difference was seen in Th1 or Th17 differentiation in CD4^+^ T cells (Fig. 3b). Meanwhile, regulatory T cells (CD4^+^FOXP3^+^; Tregs) were thought to have a tumor-promoting function in CRC,^28^ IL-37 has been found to induce Tregs in skin contact hypersensitivity model and atherosclerotic model.^29^ However, Treg cells were present in the colons of both AOM/DSS-treated IL-37tg and WT mice with no significance (Fig. 3b). In addition, no differences were noted in the number of B cells (CD19^+^), NK cells (NK1.1^+^), and dendritic cells (CD11C^+^ MHC-II^+^) (Fig. 3c, d). Myeloid-derived suppressor cells (MDSCs) can dominate tumor immunosuppressive microenvironment, it has a suppressive ability on CD8^+^ or CD4^+^ T cells in AOM/DSS-induced CAC.^30,31^ However, we did not observe significantly different levels of CD11B^+^Gr-1^+^ MDSCs in tumor-bearing colon tissue of IL-37tg mice and WT mice (Fig. 3d). In light of the DCs-dependent activation of CD8^+^ T cells mechanism in tumor protection,^32,33^ the colonization of DCs in tumor-bearing colon tissue of IL-37tg mice and WT mice were examined. We verified that there were no differences in the number of DCs (CD11C^+^MHC-II^+^) (Fig. 3d). IL-37tg mice had lower total macrophage accumulation (CD11B^+^F4/80^+^) compared with WT mice (Fig. 3d). These results suggested that IL-37 inhibits infiltration of CD8^+^ T cells in colon and MLN in the CAC model.

At rest state, CD8^+^ T cells exhibit similar frequency in MLN and spleen between IL-37tg mice and WT mice (Supplementary Fig. S6a, b), we did not find IL-37 expression in CD8^+^ T cells (Supplementary Fig. S6c). To further understand the dysfunction of CD8^+^ T cells in the CAC model, we compared the nature of CD8^+^ T cells in tumor-bearing colon tissue and MLN. There were obviously decreased proliferation rates and increased apoptosis rates of CD8^+^ T cells in tumor-bearing colon tissue of IL-37tg mice compared with those of WT mice, as assessed by Ki-67 and annexin V staining respectively (Fig. 3e). And a significant decrease was observed in the percentage of CD8^+^ T cells expressing CD103 (integrin associated with intestinal T cell retention), CD69 (early activation marker), CD107 (degranulation marker), or IFN-γ (effector cytokine) was observed in tumor-bearing colon tissue of IL-37tg mice compared with those of WT mice (Fig. 3e). These differences were also observed in the MLN (Fig. 3f). In addition, the presence of a low percentage of effector and/or effector memory (CD62L^neg^CD44^high^) and central memory (CD62L^+^CD44^high^) CD8 T cells was observed in MLN of IL-37tg mice compared with those in WT mice (Fig. 3f), which is linked to poor prognosis in human CRC patients.^34^ Similar to the results obtained for MC38 xenograft model, we found that the frequency of CD8^+^ T cells and CD8^+^IFN-γ^+^ T cells was significantly decreased in tumors and tumor-draining lymph nodes of IL-37tg mice compared with that of WT mice (Supplementary Fig. S6d–f). These results suggested that IL-37 inhibited the activation of CD8^+^ CTLs in colorectal cancer model.

**Fig. 4:**
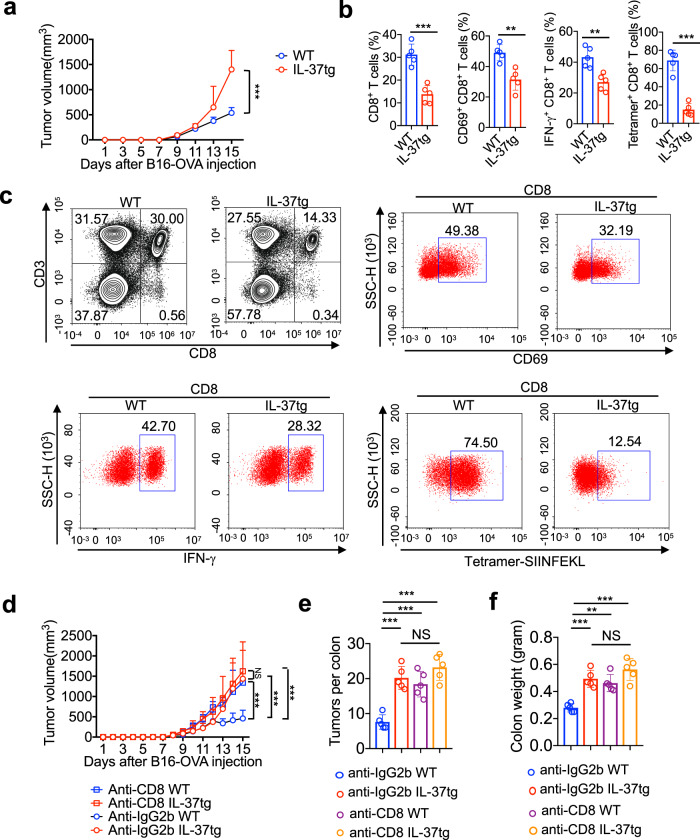
IL-37 enhanced tumorigenesis via CD8^+^ T cell inactivation. **a**–**c** IL-37tg mice and WT mice were subcutaneous injection of 5 × 10^5^ B16-OVA melanoma cells. **a** Xenograft tumor growth curve of different groups. *n* = 5/group. **b**, **c** The percentage of CD8^+^ T cells was measured 15 days after tumor inoculation. Quantification (**b**) and representative (**c**) of FACS analysis of CD8^+^ T cells and Tetramer-SIINFEKL CD8^+^ T cells, CD8^+^ T cells expressing activation marker CD69 and effector molecules IFN-γ. *n* = 5/group. **d** Xenograft tumor growth curve of different treatment groups. The mice were subcutaneous injection of 5 × 10^5^ B16-OVA melanoma cells, subsequently intraperitoneal injection of 250 μg CD8-neutralizing antibody or IgG2b isotype control twice a week, B16-OVA tumor growth was monitored. *n* = 6/group. **e**, **f** IL-37tg mice and WT mice were treated with or without AOM/DSS for 70 days, simultaneously intraperitoneal injection of 250 μg CD8-neutralizing antibody or IgG2b isotype control twice a week. **e** Quantification of the number of tumors in the colon, *n* = 5/group. **f** Colon weight was determined, *n* = 5/group. The data are presented as mean ± SD. Statistics analyzed by Two-way ANOVA analysis of variance with Turkey’s post hoc test (**a**, **d**), Two-tailed Student’s *T*-test (**b**), and One-way ANOVA analysis of variance with Turkey’s post hoc test (**e**, **f**). **P < 0.01; ***P < 0.001. NS, not significant

To test whether IL-37 is involved in specific immune responses elicited by tumors, we utilized the highly aggressive B16-OVA cancer model which overexpresses the surrogate tumor antigen, ovalbumin (OVA). The pro-tumor activity was found in IL-37tg mice compared with that in control groups (Fig. 4a). The frequency of total CD8^+^ T cells was lower from IL-37tg mice compared with that of control groups (Fig. 4b, c). Moreover, CD8^+^ T cells from IL-37tg mice exhibited decreased expression of CD69 and IFN-γ, frequency of OVA-specific CD8+ T (Tetramer^+^CD8^+^) cells was lower in IL-37tg mice compared with that of control groups (Fig. 4b, c), these results indicated that IL-37 inhibits the functional efficacy of tumor-specific CD8 T cells.

To better understand the impaired antitumor effect of IL-37 was due to depletion of CD8^+^ cytotoxic T cells, neutralizing CD8 antibodies was used. In B16-OVA tumor model and AOM/DSS model, we observed no significant differences in tumor size or numbers between WT and IL-37tg mice when injected with CD8-blocking antibodies (Fig. 4d–f), suggesting that CD8 neutralization eliminated the differences in tumor size or numbers between WT and IL-37tg mice. In conclusion, these results demonstrated the role of IL-37 in driving pro-tumor immunity by cytotoxic T cell dysfunction.

### IL-37 limited IL-18–induced functional activities of CD8^+^ T cells via SIGIRR

**Fig. 5:**
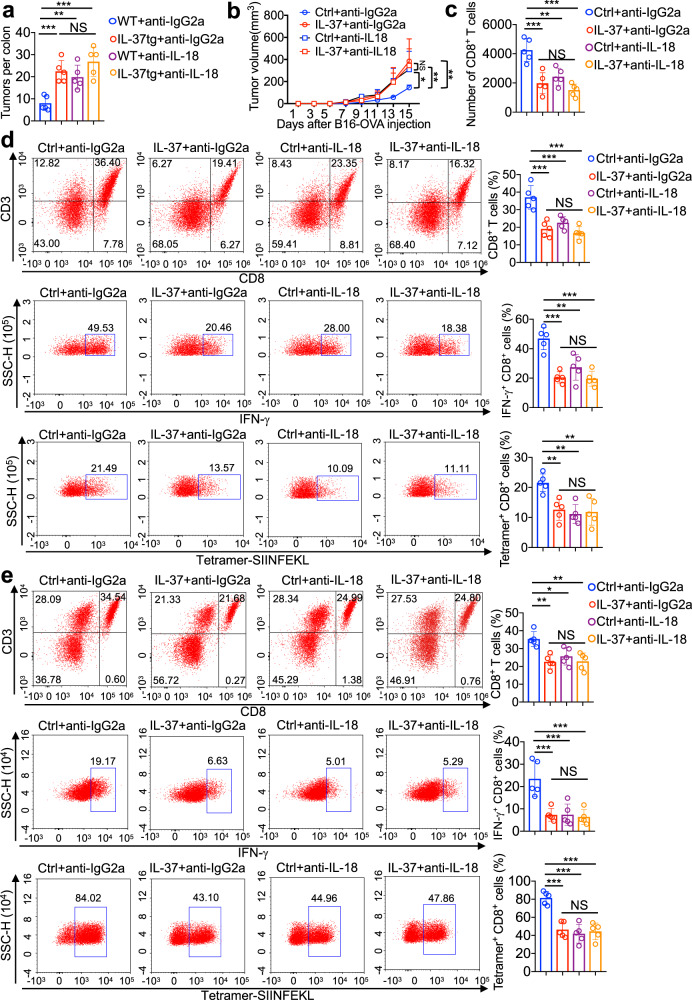
IL-37 inhibited the CD8^+^ cytotoxic T cells–mediated antitumor immunity that was depended on IL-18. **a** IL-37tg mice and WT mice were treated with or without AOM/DSS for 70 days, simultaneously intraperitoneal injection of 50 μg IL-18–neutralizing antibody or IgG2a isotype control twice a week. Quantification of the number of tumors in the colon, *n* = 5/group. **b** Xenograft tumor growth curve of different treatment groups. The mice were subcutaneous injection of 2 × 10^5^ B16-OVA melanoma cells, subsequently intravenous injection of 100 ng recombinant IL-37 every day, and intraperitoneal injection of 50 μg IL-18–neutralizing antibody or IgG2a isotype control twice a week, B16-OVA tumor growth was monitored. *n* = 5/group. **c** IL-37tg mice and WT mice were treated with or without AOM/DSS for 70 days, simultaneously intraperitoneal injection of 50 μg IL-18–neutralizing antibody or IgG2a isotype control twice a week, absolute cell numbers of CD8^+^ T cells in the tumor-bearing colon tissue as assessed by flow cytometry. A total of 200,000 live colonic cells were acquired to normalise the baselines for all samples, *n* = 5/group. **d**, **e** The mice were subcutaneous injection of 2 × 10^5^ B16-OVA melanoma cells, subsequently intravenous injection of 100 ng recombinant IL-37 every day, and intraperitoneal injection of 50 μg IL-18–neutralizing antibody or IgG2A isotype control twice a week. The percentage of CD8^+^ T cells was measured 15 days after tumor inoculation. Representative and quantification of FACS analysis of CD8^+^ T cells and Tetramer-SIINFEKL CD8^+^ T cells, CD8^+^ T cells expressing activation marker effector molecules IFN-γ in the tumor tissue (**d**) and tumor-draining lymph node (**e**). *n* = 5/group. The data are presented as mean ± SD. Statistics analyzed by One-way ANOVA analysis of variance with Turkey’s post hoc test (**a**, **c**–**e**). Two-way ANOVA analysis of variance with Turkey’s post hoc test (**b**). *P < 0.05; **P < 0.01; ***P < 0.001. NS, not significant

IL-18, which binds the IL-18 receptor 1 (IL-18Rα), synergistically enhances IFN-γ secretion from CD8 T cells,^35^ it plays an essential role in protection against colorectal tumor development.^10,12^ The expression of IL-18 was increased in colon of CAC model (Supplementary Fig. S7a). We found that the number of colonic tumors and the size of B16-OVA tumors in IL-37 absent and present mice when injected with IL-18-blocking antibodies was not significantly differences (Fig. 5a, b and Supplementary Fig. S7b). Moreover, the number or frequency of CD8^+^ T cells was not significantly differences between IL-37 absent and present mice when injected with IL-18-blocking antibodies (Fig. 5c–e), the similar results were found in frequency of IFN-γ^+^CD8^+^ cells and Tetramer^+^CD8^+^ cells (Fig. 5d, e and Supplementary Fig. S7c, d). And recombinant IL-18 did not rescue tumor burden and CD8^+^ T cells inactivation in IL-37tg mice (Supplementary Fig. S8). These results suggested that blocking IL-18 can eliminate the differences in functional efficacy of tumor-specific CD8 T cells between IL-37 absent and present mice.

**Fig. 6:**
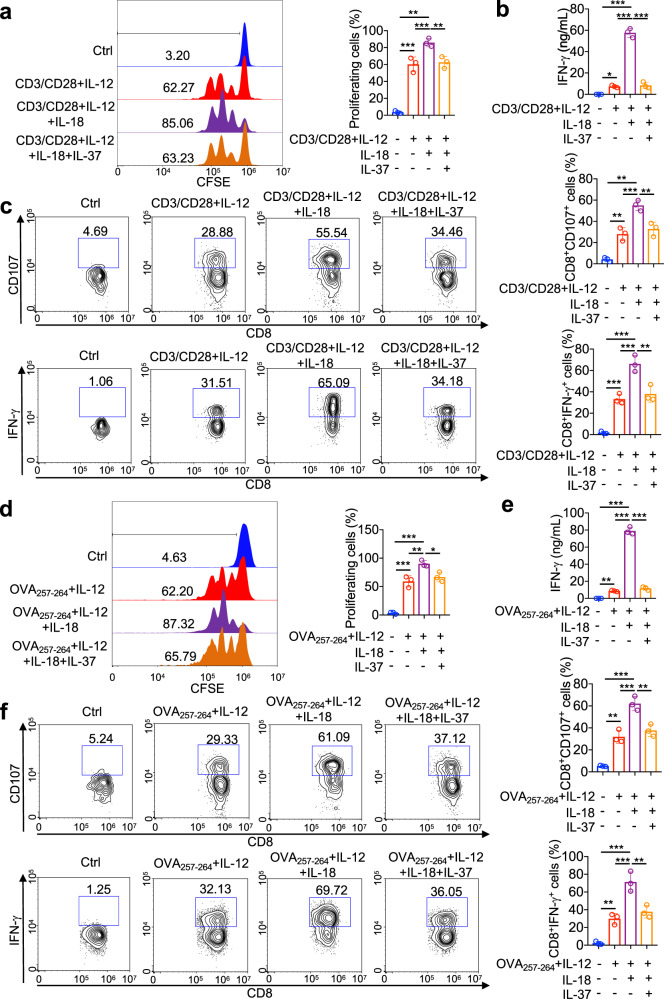
IL-37 limited IL-12/18 induce the cytotoxic activity of CD8^+^ T cells. **a** Isolated mouse naive CD8^+^ T cells were labeled with 5 μM CFSE and pulsed with or without CD3/CD28 in the presence or absence of 1 ng/mL IL-12, 100 ng/mL IL-18, 100 ng/mL IL-37b for 72 h. Proliferation was determined by the CFSE dilution assay. Representative histograms of CFSE dilution (left) and statistical analysis of proliferating cells (right). Numbers in the histogram plots represent the percentage of proliferating cells, *n* = 3/group. **b** ELISA analysis of IFN-γ levels in culture medium from isolated mouse naive CD8^+^ T cells treated with or without CD3/CD28 in the presence or absence of 1 ng/mL IL-12, 100 ng/mL IL-18, 100 ng/mL IL-37b for 72 h. *n* = 3/group. **c** Isolated mouse naive CD8^+^ T cells were treated with or without CD3/CD28 in the presence or absence of 1 ng/mL IL-12, 100 ng/mL IL-18, 100 ng/mL IL-37b for 72 h. Representative and quantification of FACS analysis of CD107^+^CD8^+^ cells and IFN-γ^+^CD8^+^ cells, *n* = 3/group. **d** Isolated mouse naive OT-I CD8^+^ cells were labeled with 5 μM CFSE and pulsed with or without 5 μg/mL OVA257-264 peptides in the presence or absence of 1 ng/mL IL-12, 100 ng/mL IL-18, 100 ng/mL IL-37b for 72 h. Proliferation was determined by the CFSE dilution assay. Representative histograms of CFSE dilution (left) and statistical analysis of proliferating cells (right). Numbers in the histogram plots represent the percentage of proliferating cells, *n* = 3/group. **e** ELISA analysis of IFN-γ levels in culture medium from isolated mouse naive CD8^+^ T cells treated with or without 5 μg/mL OVA257-264 peptides in the presence or absence of 1 ng/mL IL-12, 100 ng/mL IL-18, 100 ng/mL IL-37b for 72 h. *n* = 3/group. **f** Isolated mouse naive CD8^+^ T cells were treated with or without 5 μg/mL OVA257-264 peptides in the presence or absence of 1 ng/mL IL-12, 100 ng/mL IL-18, 100 ng/mL IL-37b for 72 h. Representative and quantification of FACS analysis of CD107^+^CD8^+^ cells and IFN-γ^+^CD8^+^ cells. *n* = 3/group. All data are presented as mean ± SD. Statistics analyzed by One-way ANOVA analysis of variance with Turkey’s post hoc test. **P < 0.01; ***P < 0.001

**Fig. 7:**
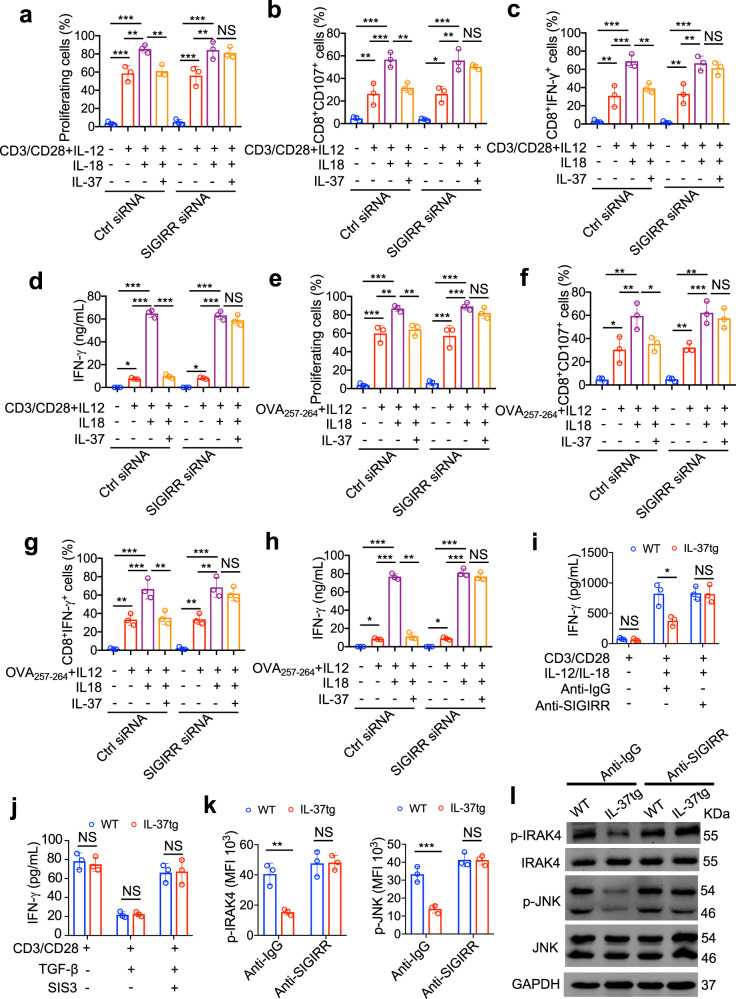
IL-37 limited IL-12/IL-18–induced functional activities of CD8^+^ T cells via SIGIRR. **a** Isolated mouse naive CD8^+^ T cells were transfected for 72 h with the SMARTpool siRNA reagent against SIGIRR or with a control Accell nontargeting siRNA, transfected CD8^+^ T cells were labeled with 5 μM CFSE and pulsed with or without CD3/CD28 in the presence or absence of 1 ng/mL IL-12, 100 ng/mL IL-18, 100 ng/mL IL-37b for 72 h. Proliferation was determined by the CFSE dilution assay. Statistical analysis of proliferating cells, numbers in the histogram plots represent the percentage of proliferating cells, n = 3/group. **b**–**d** SIGIRR siRNA or Ctrl siRNA transfected CD8^+^ T cells were treated with or without CD3/CD28 in the presence or absence of 1 ng/mL IL-12, 100 ng/mL IL-18, 100 ng/mL IL-37b for 72 h. Quantification of FACS analysis of CD107^+^CD8^+^ cells (**b**) and IFN-γ^+^CD8^+^ cells (**c**), ELISA analysis of IFN-γ levels in culture medium (**d**). *n* = 3/group. **e** Isolated mouse naive CD8^+^ OT-I cells were transfected for 72 h with the SMARTpool siRNA reagent against SIGIRR or with a control Accell nontargeting siRNA, transfected CD8^+^ T cells were labeled with 5 μM CFSE and pulsed with or without 5 μg/mL OVA257-264 peptides in the presence or absence of 1 ng/mL IL-12, 100 ng/mL IL-18, 100 ng/mL IL-37b for 72 h. Proliferation was determined by the CFSE dilution assay. Statistical analysis of proliferating cells, numbers in the histogram plots represent the percentage of proliferating cells, *n* = 3/group. **f**–**h** SIGIRR siRNA or Ctrl siRNA transfected CD8^+^ T cells were treated with or without 5 μg/mL OVA257-264 peptides in the presence or absence of 1 ng/mL IL-12, 100 ng/mL IL-18, 100 ng/mL IL-37b for 72 h. Quantification of FACS analysis of CD107^+^CD8^+^ cells (**f**) and IFN-γ^+^CD8^+^ cells (**g**), ELISA analysis of IFN-γ levels in the culture medium (**h**), *n* = 3/group. **i** Naive CD8^+^ T cells were isolated from the spleen of IL-37tg mice and WT mice, isolated CD8^+^ T cells were treated with CD3/CD28 in the presence or absence of 1 ng/mL IL-12, 100 ng/mL IL-18, 10 µg/ml anti-SIGIRR antibody, 10 µg/ml Ctrl antibody for 48 h. ELISA analysis of IFN-γ levels in the culture medium. *n* = 3/group. **j** Naive CD8^+^ T cells were isolated from the spleen of IL-37tg mice and WT mice, isolated CD8^+^ T cells were treated with CD3/CD28 in the presence or absence of 5 ng/ml TGF-β, 3 μM SIS3 for 48 h. ELISA analysis of IFN-γ levels in the culture medium. *n* = 3/group. **k**–**l** Naive CD8^+^ T cells were isolated from the spleen of IL-37tg mice and WT mice, isolated CD8^+^ T cells were stimulated with CD3/CD28, 1 ng/mL IL-12, and 100 ng/mL IL-18 presence 10 µg/ml anti-SIGIRR antibody or 10 µg/ml Ctrl antibody for 48 h. **k** Quantification of FACS analysis of IRAK4, and JNK phosphorylation in CD8^+^ T cells. MFI, mean fluorescent intensity. *n* = 3/group. **l** Immunoblotting was performed to detect phosphorylation of IRAK4 and JNK in CD8^+^ T cells. GAPDH was used as a loading control. All data are presented as mean ± SD. Statistics analyzed by One-way ANOVA analysis of variance with Turkey’s post hoc test (**a**–**h**). Statistics analyzed by Two-tailed Student’s *T*-test (**i**–**k**). *P < 0.05; **P < 0.01; ***P < 0.001. NS, not significant

The silencing of IL-18Ra impairs IL-37 activity in LPS-stimulated monocytes, consistent with the tripartite complex composed of IL-37, SIGIRR, and IL-18Ra is indispensable for the function of IL-37.^16^ SIGIRR expression was identified in CD8^+^ T cells (Supplementary Fig. S9). Therefore, we suppose whether IL-37 can antagonize IL-18 via SIGIRR, thereby dampened the effector function of CD8^+^ T cells. IL-12 exhibited a synergistic effect with IL-18 on IFN-γ production by T cell due to that IL-12 can induce IL-18Rα expression and IL-18 cannot induce IFN‐γ secretion of CD8^+^ T cells alone.^36,37^ We found that IL-18 promoted CD8^+^ T cell expansion, increased CD107 expression, and IFN-γ production in CD8^+^ T cells in presence of the IL-12 and anti-CD3/CD28, and recombinant IL-37 significantly dampened this role of IL-18 on CD8^+^ T cells (Fig. 6a–c and Supplementary Fig. S10). Similar results were obtained in OVA257-264 stimulated OT-I cells (Fig. 6d–f Supplementary Fig. S11). These findings suggested that IL-37 functionally inhibits IL-18–induce functional activities of CD8^+^ T cells. Previous research has reported that SIGIRR is a co-receptor of IL-18Rα for IL-37, serving as the checkpoint for maturation of NK cells to participate in tumorigenesis.^38^ We further investigate whether IL-37 antagonizes IL-18–induced CD8^+^ T cell expansion and effector functions depending on SIGIRR. The results show that the SIGIRR knockdown nearly eliminated the inhibited role of recombinant IL-37 on CD8^+^ T cell expansion as well as the expression of cytotoxic effector molecules (CD107 and IFN-γ) in CD8^+^ T cells in the presence of anti-CD3/CD28, IL-12, and IL-18 (Fig. 7a–d and Supplementary Fig. S9). The experiment was performed in OVA257-264 stimulated OT-I cells, with similar results (Fig. 7e–h and Supplementary Fig. S9). Compared with IL-37tg cytotoxic T cells, IFN-γ production was more sustained in WT cytotoxic T cells stimulated by IL-12 and IL-18 combinations (Fig. 7i). Importantly, restraining on effector function in IL-37tg cytotoxic T cells was abolished by SIGIRR blockade, IL-37tg cytotoxic T cells showing comparable IFN-γ production with WT cytotoxic T cell when in presence of anti-SIGIRR antibody (Fig. 7i). These data demonstrated that the inactivation of IL-37 on cytotoxic T cell effector function was dependent on SIGIRR.

Cytoplasmic IL-37 translocates to the nucleus and reduces inflammatory response via Smad3.^16,39^ TGF-β/Smad3 also can regulate CTL functions during tumor immune escape.^40^ We observed that TGF-β inhibited the secretion of IFN-γ to a similar extent in splenic CD8^+^ T cells isolated of IL-37tg and WT mice activated by αCD3/αCD28-conjugated beads (Fig. 7j), demonstrating that IL-37 participating in CD8^+^ T cell dysfunction does not seem to depend on TGF-β/Smad3 signaling pathway.

IL-18–IL-18R–MyD88 signaling has been shown to trigger the recruitment of TIR domain-containing IRAK4, leading to the activation of JNK that promote CD8^+^ T cell-mediated tumor immune surveillance.^41–44^ Moreover, SIGIRR inhibited IL-18-induced activation of IRAK4 and JNK protein kinases signaling in NK cells.^38^ In present study, we explored molecular signaling downstream exerted by IL-37, IRAK4, and JNK phosphorylation in cytotoxic T cells was assessed by flow cytometry and western blotting. Indeed, phospho-IRAK4 and phospho-JNK were decreased in IL-12/IL-18–stimulated IL-37tg cytotoxic T cells compared with that of IL-12/IL-18–stimulated WT cytotoxic T cells (Fig. 7k, l), indicating unharnessed early signaling downstream of MyD88. When blocking with anti-SIGIRR antibody, IRAK4, and JNK phosphorylation in IL-37tg cytotoxic T cells were boosted to a similar level with WT cytotoxic T cells (Fig. 7k, l). Altogether, these results demonstrate that IL-37 inactivated cytotoxic T cells function through SIGIRR-mediated IRAK4/JNK downstream signaling.

### IL-37 was highly expressed in human CRCs and predicted poor prognosis

**Fig. 8:**
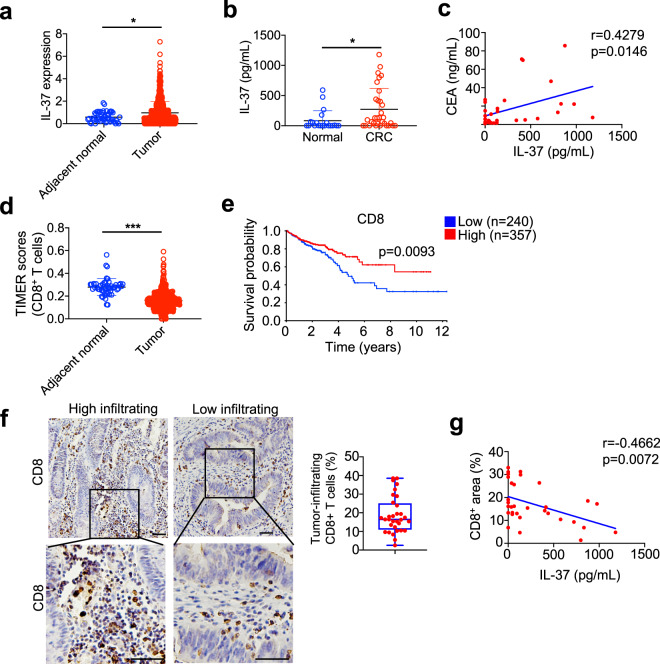
IL-37 levels were negatively correlated with CD8^+^ cytotoxic T cells in patients with CRC. **a** The expression distribution of IL-37 in CRC tissues (*n* = 620) and adjacent normal tissues (*n* = 51) from TCGA database. **b** Serum levels of IL-37 were measured by ELISA, IL-37 levels in CRC patients (*n* = 32) compared with normal controls (*n* = 21). **c** A significant correlation was found between the serum levels of IL-37 and CEA in CRC patients (*n* = 32). **d** The score distribution of CD8^+^ T cells in CRC tissues (*n* = 620) and adjacent normal tissues (*n* = 51) from TCGA database. **e** Kaplan–Meier survival curves comparing the high (red) and low (blue) expression of CD8 in colorectal cancer, the data available from the human protein atlas datasets (v 20.0.proteinatlas.org, https://www.proteinatlas.org/ENSG00000153563-CD8A/pathology/colorectal+cancer). **f** Representative pictures for low and high CD8^+^ cells infiltration in colorectal cancer tumors of CRC patients, scale bar: 50 μm. Quantification of the infiltration percentage of CD8^+^ T cells in colorectal cancer tumors of CRC patients (*n* = 32). **g** A significant correlation was found between the serum levels of IL-37 and CD8^+^ cells in colorectal cancer tumors in CRC patients (*n* = 32). The data are presented as mean ± SD, statistics analyzed by Two-tailed Student’s *T*-test, *P < 0.05. (**a**, **b**, **d**). The *r* and *p* values obtained with Spearman’s rank correlation test (**c**, **g**)

To evaluate the clinical significance of IL-37 in CRC, IL-37 expression was analyzed based on the Cancer Genome Atlas (TCGA) database, IL-37 mRNA expression was increased in CRC tissues compared to adjacent normal tissues (Fig. 8a). Moreover, we tested the IL-37 expression in the serum of 32 CRC patients and 21 healthy controls. Significantly higher serum IL-37 levels were observed in CRC patients (Fig. 8b). Moreover, the serum IL-37 level was positively correlated with serum level of typical CRC marker CEA in CRC patients (Fig. 8c). These findings indicated that increased level of IL-37 was a frequent event in CRC patients.

**Fig. 9:**
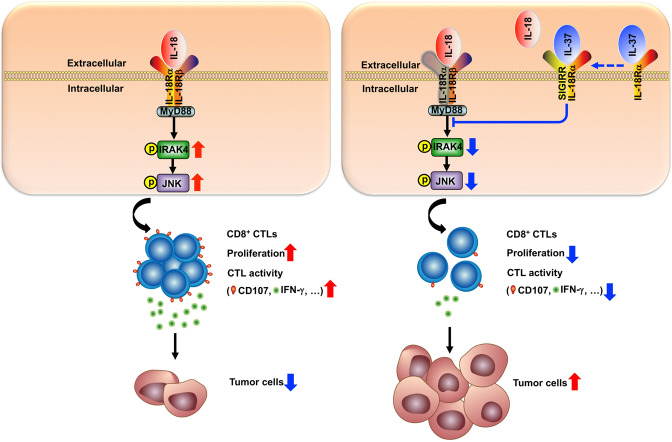
Schematic representation for the functional mechanism of IL-37 in CRC. IL-37 requires SIGIRR to interrupts the IL-18 drives MyD88–IRAK4–JNK signaling in CD8^+^ CTLs, subsequently leads to suppression of proliferation and cytotoxic activity of CD8^+^ CTLs, IFN-γ production are decreased. Dysfunctional CD8^+^ CTLs were deprived of immune surveillance for tumor, enhancing tumorigenesis of colorectal cancer.

The mouse experiments of our study suggested that IL-37 impaired CD8^+^ T cell-mediated antitumor effect. The tumor-infiltrating CD8^+^ T cells were analyzed by TIMER2.0 (http://timer.cistrome.org/), we found that immune infiltration levels of CD8^+^ T cells for TCGA were decreased in CRC tissues compared with that of adjacent normal tissues (Fig. 8d). Moreover, Kaplan–Meier survival curves for CD8 from Human Protein Atlas Dataset^45^ (http://www.proteinatlas.org) show that CRC patients with low CD8 expression levels in their primary tumors exhibited a significantly poorer prognosis (Fig. 8e). We, therefore, correlated IL-37 serum level with CD8^+^ tumor-infiltrating lymphocytes to determine whether IL-37 expression could predict cytotoxic T cell potential. IHC staining were performed to detect CD8^+^ T cells in CRC tissues, the CD8^+^ T cell infiltration in CRC tissues were illustrated in Fig. 8f. We observed a negative correlation between serum IL-37 level and CD8^+^ T cell infiltration in CRC patients (Fig. 8g). These findings strengthen the connections between IL-37 and cytotoxic CD8^+^ T cells in CRC patients, and specifically explain the tumor promotion roles of IL-37 (Fig. 9).

## Discussion

Tumor development are strongly influenced by innate and adaptive immunity, which either promote or attenuate tumorigenesis. Chronic inflammation promotes tumor development, progression, and metastasis. However, tumor development and malignant progression are also associated with loss of normal immune regulatory processes, which activate antitumor immune responses. Emerging studies has revealed the key regulatory effect of IL-1 family cytokines (IL-18, IL36, IL-33 et al.) in CD8^+^ CTL–mediate antitumor immune responses.^9,21^ IL-18 has been identified as key agonist of CD8^+^ CTL activation to induced IFN-γ production.^9^ The powerful activity of IL-18 agonism in mice tumor model have emphasize the great attraction of target IL-18 pathway for tumor immunotherapy.^12^ Experimental studies have shown that IL-36 and IL-33 can facilitate adaptive tumor antigen-specific CD8^+^ CTL immune response, and effectively control tumor escape.^46,47^ However, the role of IL-37, an new member of IL-1 family, for CD8^+^ CTL tumor immunosurveillance is not well understood, although IL-37 protects from carcinogenesis through suppress of chronic inflammation during inflammation-cancer transformation.^22^ Here, we revealed the tumorigenic effects of IL-37 in CRC, and first uncovered the tumorigenic mechanism of IL-37 via CD8 dysfunction, our findings highlight the great potential of immunotherapeutic intervention for targeting IL-37/SIGIRR signaling.

Inflammatory bowel disease is closely related to the colorectal carcinogenesis. Disruption of the intestinal epithelial barrier can initiate chronic inflammation of colitis, which is regarded to responsible for carcinomatous change of intestinal epithelium. Cytokines and inflammatory cells can constitute a complex immune network, which affects tumor progression by regulating the proliferation, apoptosis, and invasion of intestinal epithelial cells.^48^ Previous studies have reported that IL-37 have anti-inflammation activity in experimental colitis, it inhibits the expression of TNF and IL-1β and increased the production of IL-10, and decreased leukocyte recruitment to the colon.^23^ Consistent with these findings, the anti-inflammation properties of IL-37 was observed in colitis and colitis-associated colorectal cancer in our study, IL-37 inhibits the infiltration of immune cells, such as CD8^+^ T cells and macrophage, in the intestine. Compared with the previous transgenic mouse by McNamee et al., constructed IL-37tg mice in our study exhibit different expression patterns, especially overexpression of IL-37 in colonic tissue. This may be more closely match IL-37 expression patterns in homo sapiens, because previous studies indicated that IL-37 was expressed in intestinal epithelial cells and as well as inflammatory cells in human colon tissues.^49^ Our findings have shown that IL-37 can inhibit IL-18-mediated signaling. In DSS model, IL-18 secretion was significantly induced,^48,50^ and IL-18 can mediate intestinal epithelial cell proliferation and tissue repair following DSS injury, and thereby leads to attenuates symptoms of colitis.^48,50–52^ For IL-37tg mice, we found that the expression level of IL-37 was significantly higher in colon after treatment with DSS than steady state. Therefore, more severe colitis symptoms were detected in IL-37tg mice, perhaps because abundant IL-37 in colon inhibited IL-18–mediated cytoprotective effects in the DSS model. In tumor microenvironment, immune system has capability to either obstruct tumor progression or promote carcinomatous change, tumor development, and metastasis. Which fate depends on balance trend between antitumor factors and tumor-promoting factors involved in innate and adaptive immunity.^53^ CD8^+^ T cell serves as primary effector cells indispensable for disrupting tumors. In colorectal cancer model, we showed that suppressed immune status and inactivated CD8^+^ T cells in IL-37tg mice, thus responsible for cancer susceptibility of IL-37.

IL-37 has a dual anti-inflammatory function, it can be secretion from diverse cells and play an anti-inflammatory effect. In addition, intracellular IL-37 is cleaved and matured by caspase-1, enters the nucleus through Smad3-dependent pathway, and further regulates the nuclear activity of Smad3. We inspect the extracellular and intracellular pathways employed by IL-37 in the present research. Nevertheless, TGF-β/Smad3 signaling seemed not to engage in IL-37-mediated T cell dysfunction. Molgora et al. reported that SIGIRR knockout unleashes resistance to liver cancer via NK cells, suggesting that SIGIRR was a checkpoint of NK cell function.^38^ Evidenced by the abolition of restraining on cytotoxic T cell effector function and reversion of MyD88 downstream JNK and mTOR signal activation in IL-37tg cytotoxic T cells by SIGIRR blockade, we concluded that cytotoxic T cells dysfunction of IL-37 was regulated by SIGIRR-mediated IL-1 receptor family signaling blockade, the tripartite complex IL-37–SIGIRR–IL-18Ra formation is crucial for the inhibitory properties of IL-37 on antitumor immunity, consistent with a previous report.^16^ Moreover, this cytokine-cytokine receptor interaction may be the main effect pattern of secretory factor IL-37.

IL-18 known as IFN-γ inducing factor enhances antitumor immunity in various cancer types as previously reported.^54^ However, clinical administration of recombinant IL-18 alone has been curtailed due to its lack of strong efficacy in clinical trials,^55^ the level of IL-37 should be considered in this therapeutic strategy. IL-37 was highly expressed in human CRCs and positive corrected with CRC biomarker CEA levels, and therefore immunotherapeutic intervention for IL-37/SIGIRR pathway is necessary to CRC immunotherapies. CD8^+^ CTLs is poorly infiltration in CRC and indicates poor prognosis, and the negative correlation between tumor infiltrated CD8^+^ CTLs and IL-37 level were illustrated in CRC. Previous studies have suggested that elevated levels of tumor-infiltrating CD8^+^ CTLs are closely related to the antitumor effects of various cancer types, including melanoma, skin cancer, colon cancer et.al.^56^ The unique mechanism of IL-37 act on cytotoxic CD8^+^ T cells provides a powerful basis for development of clinically antineoplastic agent for the different types of cancer.

In summary, this study reveals a novel crucial IL-37 mechanism involved in dampening antitumor immunity by inactivation of cytotoxic T cells in the CRC. Moreover, our data enhance knowledge regarding IL-37 and highlight the role of IL-37/SIGIRR signaling in the CRC, and these findings will aid in the development of new strategies for the treatment of colorectal cancer.

## Methods

### Patient specimens

Colon cancer tissues and serum were obtained from 32 patients with primary colon cancer during surgical CRC resection at West China Hospital. Staging was based on the American Joint Committee on Cancer (AJCC). During the diagnosis of colorectal cancer, cases were confirmed by colonoscopy and biopsy. Biopsy samples from primary CRC tumor and serum samples were obtained from CRC patients at the time of operation before any therapeutic intervention. In addition, the normal serum was obtained from 21 matched normal donors. Samples were collected from donors consecutively with standardized protocol. This study complied with the Declaration of Helsinki and was approved by the Ethics Committee of West China Hospital. Written informed consent was obtained from all study participants prior to the study. The clinical characteristics of the normal subjects and patients with CRC are provided in Supplemental Table 1.

### Animal model

The IL-37tg mice were obtained from Cyagen Biosciences Inc. Briefly, the full-length human IL-37 (IL-37b) cDNA (NM_014439.4) was cloned from IL-37b expressing pCMV6-Entry vector (Origene) and inserted downstream of the CMV promoter in pRP.ExSi vector confirmed by sequencing analysis. pRP.ExSi IL-37 expression plasmid was injected in fertilized eggs of C57BL/6N mice, and implanted into C57BL/6N females. Mouse genomic DNA was extracted from ear biopsies (Bimake), genotyping was performed by PCR assay, the primers for transgene product can amplify the 483 bp of the ranging from CMV region to IL-37 ORF with Rgs7 as internal control (632 bp). Primers used were listed as follows: transgene IL-37b (forward 5′-TGGCAGTACATCTACGTATTAGTCA-3′, reverse 5′-ATGAATGCTGAATTTCTTCGGGTT-3′) and internal control Rgs7 (forward 5′-CAACCACTTACAAGAGACCCGTA-3′, reverse 5′-GAGCCCTTAGAAATAACGTTCACC-3′). Western blotting was performed to detect IL-37 expression in PCR-positive mice with anti-IL-37 polyclonal antibody against IL-37 (R&D Systems). White blood cell counts were measured using a micro-semi CRP hematology analyzer (Axonlab). OVA-specific T cell receptor-transgenic OT-I mice were obtained from the State Key Laboratory of Biotherapy.

Colitis-associated colorectal cancer was induced by injection of mice intraperitoneally as previously reported.^57^ IL-37tg and WT littermates on C57BL/6N background (aged 8–10 weeks) were intraperitoneal injection with AOM (Sigma) dissolved in 0.9% NaCl (10 mg/kg/dose). Mice were treated with 2% DSS salt (MP Biomedicals) in drinking water at 5 days after AOM injection, then regular water for 14 days, this cycle was repeated thrice. Animals were sacrificed and tissues were harvested and analyzed. For experimental colitis model, the mice were given 3% DSS in their drinking water for 7 days, then given regular drinking water for an additional 3 days.

For the depletion of CD8^+^ T cells and IL-18, CAC mice were intraperitoneal injections with 250 μg of anti-CD8 (Bio X Cell) and anti-IgG2b isotype control (Bio X Cell) twice a week for the entire duration of the experiment. And intraperitoneal injection of 50 μg IL-18–neutralizing antibody or IgG2a isotype control twice a week for the entire duration of the experiment.

For MC38 tumor model, mice were subcutaneously inoculated with 1 × 10^6^ MC38 cells on the right flank on IL-37tg mice and WT littermates. For the recombinant IL-18 administration, intravenous injection of 20 ng recombinant IL-18 (R&D) or PBS every other day for the entire duration of the experiment. Tumor growth was measured and calculated as follows: V = (length × width^2^) × 0.5. Mice were sacrificed and tumor tissues were harvested and analyzed.

For B16-OVA tumor treatment and monitoring, mice were subcutaneously inoculated with 5 × 10^5^ B16-OVA cells on the right flank on IL-37tg mice and WT littermates. For the depletion of CD8^+^ T cells, mice were intraperitoneal injections with 250 μg of anti-CD8 (Bio X Cell) and anti-IgG2b isotype control (Bio X Cell) twice times a week for the entire duration of the experiment. Mice were subcutaneously inoculated with 2 × 10^5^ B16-OVA on the right flank on WT mice, subsequently intravenous injection of 100 ng recombinant IL-37 every day, and intraperitoneal injection of 50 μg IL-18–neutralizing antibody or IgG2a isotype control twice a week for the entire duration of the experiment. Tumor growth was monitored by measuring the perpendicular diameters of tumors, tumor volume was calculated as follows: V = (length × width^2^) × 0.5. Mice were sacrificed and murine tissues were harvested and analyzed.

For LoVo xenograft model, athymic nude mice (Balb/c-nu, male, 5-6 weeks old) were purchased from the Beijing Vital River Laboratory Animal Technology Co., Ltd (401). 5 × 10^6^ IL-37 stably transfected Lovo cells or control cells were subcutaneously injected into left flank of mice in 100 μL of PBS. Tumor growth was measured and calculated as follows: V = (length × width^2^) × 0.5. Mice were sacrificed and tumor tissues were harvested and analyzed.

Animals were sacrificed under isoflurane inhalation followed by cervical dislocation after terminal studies. All animal experiments were approved by the Committee on the Ethics of Animal Experiments of Sichuan University. The experimental procedures were conducted according to the National Institutes of Health Guide for the Care and Use of Laboratory Animals (NIH publication No 85-23).

### Cell lines

LoVo cells and B16-OVA cells and MC38 cells were deposited in the State Key Laboratory of Biotherapy. The human IL-37b gene overexpression lentivirus and control lentivirus were obtained from Shanghai GenePharma Co., Ltd. Lentiviral production was performed in LoVo cells according to the manufacturer’s instructions, the LoVo cells stably expressing the lentiviral construct were selected with 2 μg/mL puromycin (Thermo Fisher Scientific). LoVo cells, MC38 cells and B16-OVA cells were grown in DMEM (Thermo Fisher Scientific) and RPMI-1640 medium (Thermo Fisher Scientific), respectively. Cells were maintained as a monolayer in a humidified incubator, 5% CO_2_, at 37 °C in specific culture medium supplemented with 10% (v:v) fetal bovine serum (FBS; Thermo Fisher Scientific), 100 U/mL penicillin G, and 100 μg/mL streptomycin sulfate (Thermo Fisher Scientific). All cells were found to be free from mycoplasma contamination.

### T-cell Isolation and Culture

CD8^+^ T cells were isolated from the spleen using the mouse naive CD8^+^ T cell isolation kit (STEMCELL Technologies) according to the manufacturer’s instructions. For gene silencing in cultured CD8^+^ T cells, Accell mouse SIGIRR SMARTpool siRNA (Dharmacon; target sequences: CCUACGUGUCCUAUAGCGA, CCCUGCUCUAUGUUAAGUG, UCGUGGUUCUUUCAGAUGC, GGAUGAUGUGUAGCCCAUA) was applied to induce SIGIRR knockdown according to the manufacturer’s protocol, Accell non-targeting siRNA pool (Dharmacon) was applied as the control. Cells were treated with Accell siRNA resuspended in the Accell delivery media (Dharmacon). After 72 h culture, the delivery medium was replaced with normal growth medium. For CD8^+^ T cell proliferation, CD8^+^ T cells were labeled with 5 μM CFSE (CFSE Cell Division Tracker Kit, Biolegend) according to the manufacturer’s instructions. Isolated CD8^+^ T cells and OT-I CD8^+^ T cells were activated by using Dynabeads Mouse T-Activator CD3/CD28 (Thermo Fisher Scientific) and with 5 μg/mL OVA257-264 peptides (GenScript), respectively. CD8^+^ T cells were administrated with 1 ng/mL IL-12 p70 (PeproTech), 100 ng/mL IL-18 (R&D Systems), 5 ng/ml TGF-β (PeproTech) and 100 ng/mL IL-37b (R&D Systems), 3 μM SIS3(Sigma-Aldrich), 10 µg/ml anti-SIGIRR antibody (R&D Systems), 10 µg/ml Ctrl antibody (R&D Systems) in RPMI-1640 medium (Thermo Fisher Scientific) supplemented with 10% (v:v) fetal bovine serum (FBS, Thermo Fisher Scientific), 100 U/mL penicillin G and 100 μg/mL streptomycin sulfate (Thermo Fisher Scientific) for 48 or 72 h.

### Western blotting

Whole-cell lysates or tissue lysates were resolved by SDS-PAGE and transferred to PVDF membrane. Blots were probed with antibodies. After incubation with HRP-conjugated secondary antibody (ZSGB-BIO), and further detected using ECL reagents (MerckMinipore). Primary antibodies including IL-37b (R&D Systems), Bcl-xL (Cell Signalling Technology), CDK4 (Cell Signalling Technology), p-p65 (Cell Signalling Technology), c-Myc (R&D Systems), Smad3 (Cell Signalling Technology), p-Smad3C (Cell Signalling Technology), p-Smad3L (Abcam), JNK (Cell Signalling Technology), p-JNK (Cell Signalling Technology), IRAK4 (Cell Signalling Technology), p-IRAK4 (Cell Signalling Technology), GAPDH (Cell Signalling Technology), β-Actin (Cell Signalling Technology).

### Quantitative real-time PCR

RNA was isolated using the RNeasy Kit (Sigma-Aldrich) per the manufacturer’s instructions and converted into cDNA. Gene expression was assessed using 2× SYBR Green Master Mix according to the manufacturer’s instructions (Applied Biosystems). Sequences for qRT-PCR primers are listed in Supplemental Table 2. qPCR data were analyzed by the 2^–ΔΔCT^ method, with Actb as the housekeeping gene.

### Enzyme-linked immunosorbent assay (ELISA)

The 200–300 mg of colon tissue was washed in cold PBS supplemented with penicillin and streptomycin. These segments were cut into small pieces and cultured in 12-well flat bottom culture plates (Falcon) in serum-free RPMI medium. 100 U/mL penicillin G and 100 μg/mL streptomycin sulfate (Thermo Fisher Scientific) was supplemented to prevent bacteria growth. After incubation at 37 °C for 24 h, the medium was collected and examined for cytokine and chemokine production with MCP-1 ELISA kit, MIP-2 ELISA kit, IL-6 ELISA kit, TNF-α ELISA kit, KC ELISA kit, and IFN-γ ELISA kit, CCL2 ELISA kit, CXCL2 ELISA kit, CXCL8 ELISA kit, and IL-18 ELISA kit according to the manufacturer’s instruction. CXCL8 ELISA kit and IL-18 ELISA kit were purchased from Thermo Fisher Scientific, other ELISA kits were purchased from NeoBioscience. Cytokine and chemokine production is normalized by total colon tissue weight (whole-colon culture).

IL-37 ELISA kit (R&D Systems) was used to detect the levels of IL-37 in the colon tissue homogenates and human serum. IL-37 production in colon tissue homogenates is normalized by total protein amount (crypt protein lysate) measured by BCA analysis (Pierce). IFN-γ ELISA kit (BioLegend) was used to detect the levels of IFN-γ in cell culture supernatants. All assays were performed according to the manufacturers’ instructions.

### Hematoxylin and eosin (H&E) staining

Colon sections or tumor samples were fixed in 4% paraformaldehyde in PBS, embedded in paraffin, sectioned, and stained with H&E for histopathologic examination.

### Immunohistochemistry

Colon tissue or tumor tissue were fixed in 4% paraformaldehyde in PBS, and the fixed sections were incubated in 3% H_2_O_2_ solution in PBS at room temperature for 10 min. Antigen retrieval was performed in sodium citrate buffer (0.01 M, pH 6.0) in a microwave oven at 1000 W for 3 min. Nonspecific antibody binding was blocked by incubation with 5% normal goat serum in PBS for 1 h at room temperature. Slides were stained overnight at 4 °C with the following primary antibodies: Ki67 (Abcam), p-Stat3 (Cell Signalling Technology), Cleaved Caspase-3 (Cell Signalling Technology), Phosphorylated H2AX (γ-H2AX, Cell Signalling Technology), CD8 (Abcam), IL-37(Abcam), Claudin-3 (Thermo scientific).

### BrdU staining

The 5 mg/ml of BrdU in PBS was intraperitoneal injection to mice via i.p. Mice were sacrificed at 24 h after BrdU injection. The same segment of the distal colon was fixed in 10% neutral formalin and paraffin embedding. Proliferating cells were detected with the BrdU detection kit (BD Bioscience). Tissues were counterstained with hematoxylin. The number of BrdU-positive cells was quantified by the number of cells in intact, well-orientated crypts.

### TUNEL assay

Fixed sections obtained from untreated mice were fixed with 4% paraformaldehyde and permeabilized by 0.1% Triton X-100. TUNEL assay kit (Promega) was used to detect apoptotic cells and DAPI was used to stain the nuclei.

### Flow cytometry

The cells were extracted from lymph nodes, spleen, or colon tissue digested by collagenase. Isolated cells were stained with following antibodies: CD8-PE-CY7 (Thermo Scientific), CD4-APC-Fire™ 750 (BioLegend), IFN-γ-FITC (BioLegend), IL-17-APC (BioLegend), FOXP3-PE (BioLegend), CD19-PE (BioLegend), NK1.1-FITC (BioLegend), CD11B-APC (BioLegend), Gr-1-FITC (BioLegend), F4/80-PE (BioLegend), CD11C-APC-CY7 (BioLegend), MHC-II-PE/Dazzle™ 594 (BioLegend), CD8-APC (BioLegend), Ki67-PerCP-CY5.5 (BioLegend), Annexin V-FITC (BioLegend), CD103-PE (BioLegend), CD69-PE-CY7 (BioLegend), CD44-PE-CY7 (BioLegend), CD62L-FITC (BioLegend), CD107-FITC (BioLegend), IFN-γ-APC (BioLegend), CD107-PE (BioLegend), IFN-γ-PE (BioLegend), CD3-FITC (BioLegend), CD3-APC/Fire™ 750 (BioLegend), CD8-APC (BioLegend), IFN-γ-PE-CY7 (BioLegend), CD69-APC/Fire™ 750 (BioLegend), Tetramer-SIINFEKL-PE (MBL), p-JNK-PE (Cell Signaling Technology), p-IRAK4-Alexa Fluor 488 (Cell Signaling Technology). Intracellular staining was carried out using the Cytofix/Cytoperm kit (BD Pharmingen) following a 4 h restimulation by PMA/ionomycin (Sigma) in the presence of GolgiPlug (BD Pharmingen). Flow cytometric data acquisition was performed on a NovoExpress flow cytometer and analyzed with NovoExpress software (ACEA Biosciences).

### Analysis of TCGA data

Tumoral RNA-seq data were downloaded from The Cancer Genome Atlas (TCGA) dataset (https://portal.gdc.cancer.gov/) in January 2020. Human IL-37 mRNA differentiated expression in CRC versus adjacent normal tissues, correlation between IL-37 with CD107 data for CRC were analyzed. To make reliable immune infiltration estimations, we utilizes the immunedeconv, an R package with TIMER algorithm.^58^

### Statistical analysis

All statistical analysis was performed with GraphPad Prism 8 software (GraphPad Software Company, version 8.0.0). Student’s *t*-test was used for comparing two groups, one-way and two-way ANOVA analysis of variance with Turkey’s post hoc test was utilized for the comparisons of multiple comparison experiment. Spearman’s rank correlation test was used to analyze the relationship between two quantitative variables. Kaplan–Meier method was used for survival analysis. All data are presented as mean ± SD. *P* < 0.05 was considered statistically significant.

## Supplementary information

Revised Supplementary Materials - Marked Up

Revised Supplementary Materials - clean version
