# Supplementary material for: Interleukin-37 promotes colitis-associated carcinogenesis via SIGIRR-mediated cytotoxic T cells dysfunction
Source: Signal Transduct Target Ther. 2022 Jan 20;7:19. doi: 10.1038/s41392-021-00820-z (PMC8770466; doi:10.1038/s41392-021-00820-z)
Supplement: Supplementary file 1 — Revised Supplementary Materials - Marked Up [file 41392_2021_820_MOESM1_ESM.docx]

Supplementary Materials for

Interleukin-37 Promotes Colitis-associated Carcinogenesis via SIGIRR-mediated Cytotoxic T Cells Dysfunction

Zhen Wang,^1, 2, 3^ Fan-lian Zeng,^1^ Ya-wen Hu,^1^ Xiao-yan Wang,^1^ Fu-lei Zhao,^1^ Pei Zhou,^1^ Jing Hu,^1^ Yuan-yuan Xiao,^1, 4, 5^ Zhong-lan Hu,^1^ Ming-feng Guo,^1^ Xiao-qiong Wei,^1^ Xiao Liu,^1^ Nong-yu Huang,^1^ Jun Zhang,^1^ Shu-wen Chen,^1^ Juan Cheng,^1^ Hua-ping Zheng,^1^ Hong Zhou,^1^ Qi-xiang Zhao,^1^ Chen Zhang,^1^ Yan Hao,^1^ Song Zou,^6^ Yi-yue Gui,^6^ Jia-dong Yu,^1^ Lin-na Gu,^1^ Cheng-cheng Yue,^1^ Hao-zhou Zhang,^1^ Wen-ling Wu,^1^ Yi-fan Zhou,^1^ Xi-kun Zhou,^1^ Guo-bo Shen,^1^ Xiu Teng,^1, 7, *^ and Jiong Li^1, *^

Correspondence to: Jiong Li (lijionghh@scu.edu.cn) or Xiu Teng (tengxiu@wchscu.cn)

**This PDF file includes:**

Figures. S1 to S11

Tables S1 to S2


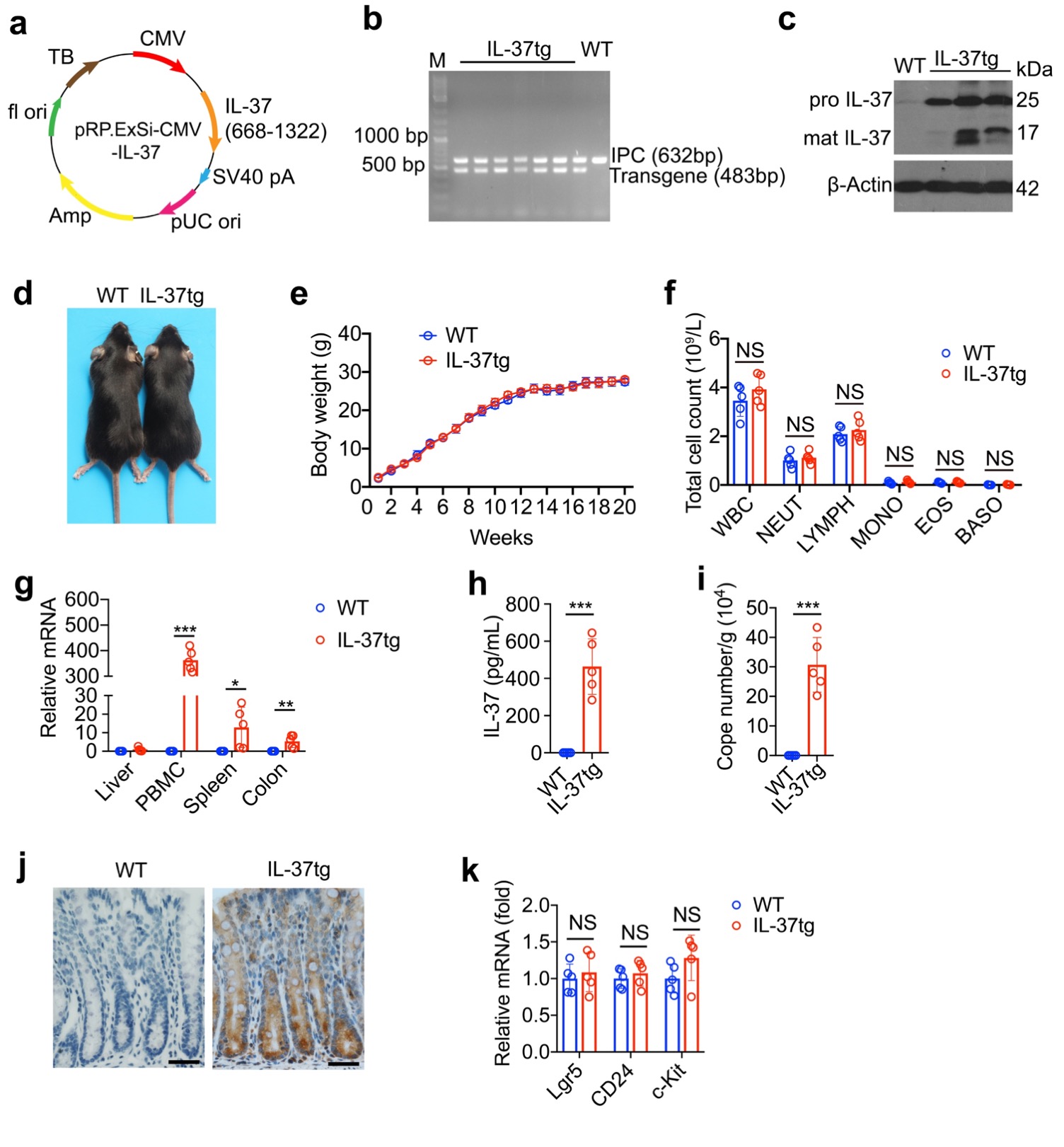


Figure. S1.

**Generation of IL-37tg mice. a** The ORF of IL-37 was inserted into vector pRP.ExSi to generate the transgenic construct. **b** PCR with the primers identifies either IL-37tg mice or WT mice, in which the product is detected as 483 bp transgene and 632 bp internal positive control (IPC). **c** Representative immunoblots of the IL-37 in IL-37tg mice and WT mice. β-Actin was used as a loading control. **d** Representative images of IL-37tg mice and WT mice. **e** Body-weight development in IL-37tg mice and WT mice, n=5/group. **f** Cells formula from blood samples collected at IL-37tg mice and WT mice, white blood cell counts were measured using a micro-semi CRP hematology analyzer. WBC, white blood cells; NEU, neutrophils; LYMPH, lymphocytes; MONO, monocytes; EOS, eosinophils; BASO, basophils. n=6/group. **g** RT-qPCR analysis was performed for IL-37 gene from IL-37tg mice and WT mice different tissues, n=5/group. **h** ELISA assessed the levels of IL-37 in colon homogenate of IL-37tg mice and WT mice, IL-37 production is normalized by total protein amount (2 mg/mL) of crypt protein lysate, n=5/group. **i** qRT-PCR analysis of transgene copy number in colon tissues of IL-37tg mice and WT mice. **j** Representative IL-37 immunohistochemistry of colon in IL-37tg mice and WT mice, scale bars: 50 μm. **k** qRT-PCR analysis of the mRNA levels of the indicated genes in colon tissues of IL-37tg mice and WT mice, n=5/group. All data are presented as Mean ± SD. Statistics analyzed by Two-tailed Student’s T-test. **P* < 0.05; ***P* < 0.01; ****P* < 0.001; NS, not significant.


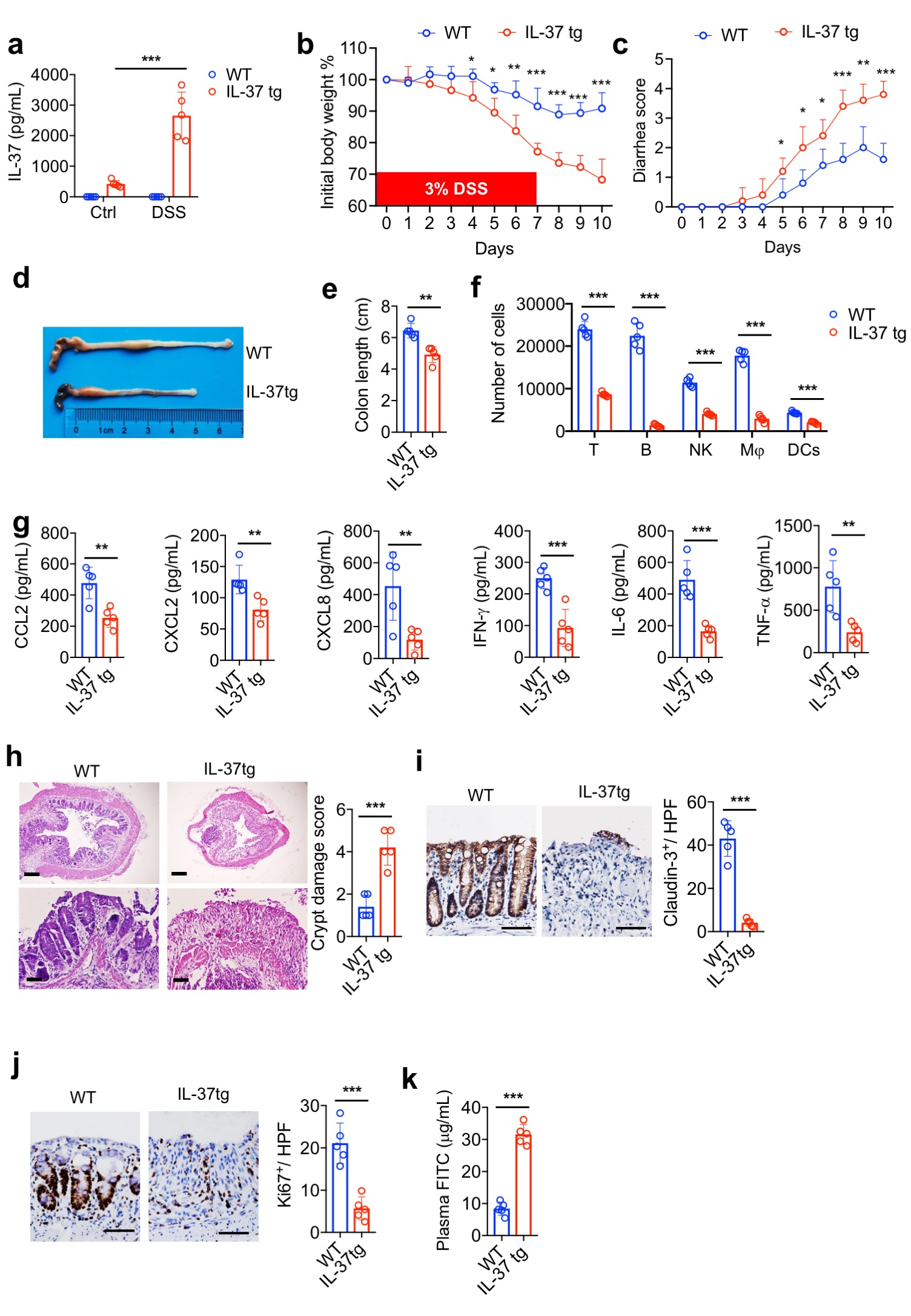


Figure. S2.

**IL-37tg mice are highly susceptible to acute dextran sulfate sodium treatment.** **a-k** WT and IL-37tg mice were given 3% DSS in their drinking water for 7 days, then given regular drinking water for an additional 3 days. **a** ELISA assessed the levels of IL-37 in colon of IL-37tg mice and WT mice treatment with or without DSS, IL-37 production is normalized by total protein amount (2 mg/mL) of crypt protein lysate, n=5/group. **b** Body weight loss of WT and IL-37tg mice. n=5/group. **c** Stool consistency and intestinal bleeding were monitored on daily basisand diarrhea was scored for each mouse. n=5/group. **d** Representative image of the colon after DSS administration. **e** Colon length of DSS-treated mice was measured. n=5/group. **f** Absolute cell numbers of CD3^+^ T cells (T cells), CD19^+^ cells (B cells), NK1.1^+^ cells (NK cells), CD11B^+^F4/80^+^ cell (Mϕ cells) and CD11C^+^MHC-II^+^ cells (DCs) in the colon tissue as assessed by flow cytometry. A total of 200 000 live colonic cells were acquired to normalise the baselines for all samples, n=5/group. **g** The colon tissue from IL-37tg mice and WT mice was cut into small pieces and incubated in serum-free RPMI medium for 24 hours. Secreted chemokines and cytokines in the medium were measured by ELISA assay, n=5/group. **h** H&Es taining of colon sections derived from WTand IL-37tg after treatment with DSS. Scale bar, 200 μm (up) and 50 μm (down). Histopathology scores evaluating the tissue involvement of crypt damage. n=5/group. **i** Representative Claudin-3 immunohistochemistry of colon, scale bars: 100 μm. Claudin-3-positive cells were quantified by counting the stained dots. n=5/group. **j** Representative Ki67 immunohistochemistry of colon, scale bars: 100 μm. Ki67-positive cells were quantified by counting the stained dots. **k** Plasma FITC-dextran concentrations in WT and IL-37tg mice, 4 hr after oral gavage of FITC-dextran (400 μg/g of body weight), are shown. n=5/group. The data are presented as Mean ± SD. Statistics analyzed by Two-tailed Student’s T-test. **P* < 0.05; ***P* < 0.01; ****P* < 0.001.


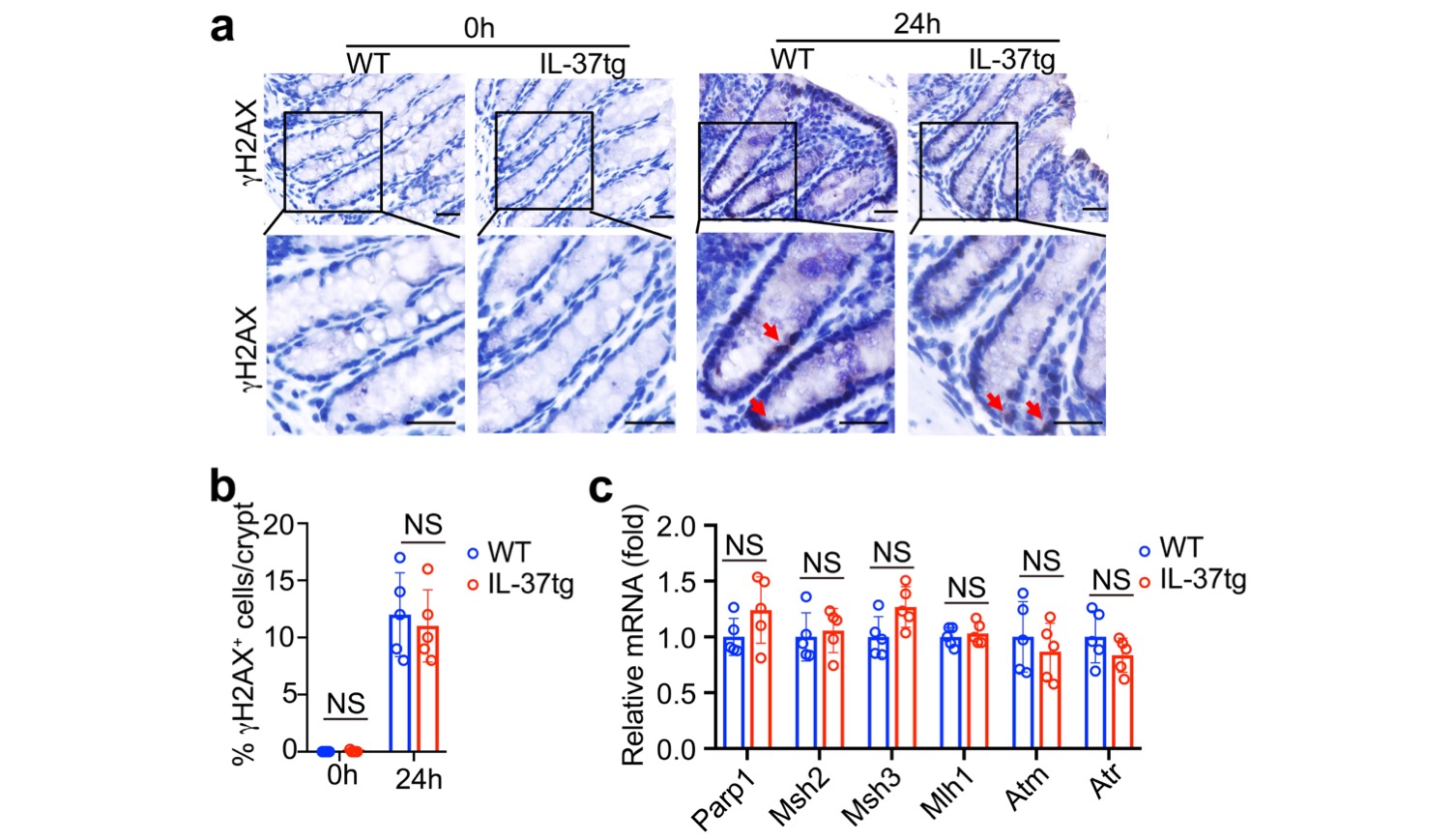


Figure. S3.

**IL-37 did not result in enhanced DNA damage initiated by AOM. a** Representative immunohistochemical staining of γH2AX in the colon of IL-37tg mice and WT mice at 0 h and 24 h after AOM administration, scale bar: 100 μm**. b** Positive cells were quantified by counting the stained dots in the colon of IL-37tg mice and WT mice at 0 h and 24 h after AOM administration, n=8/group. **c** IL37tg mice and WT mice were injected with AOM on day 0 and administered 3 rounds of 2% DSS in drinking water, the level of DNA repair gene expression was detected using real-time RT-PCR at day 70 after AOM/DSS administration. The gene expression was normalized to β-actin levels. n=8/group. All data are presented as Mean ± SD. Statistics analyzed by Two-tailed Student’s T-test. NS, not significant.


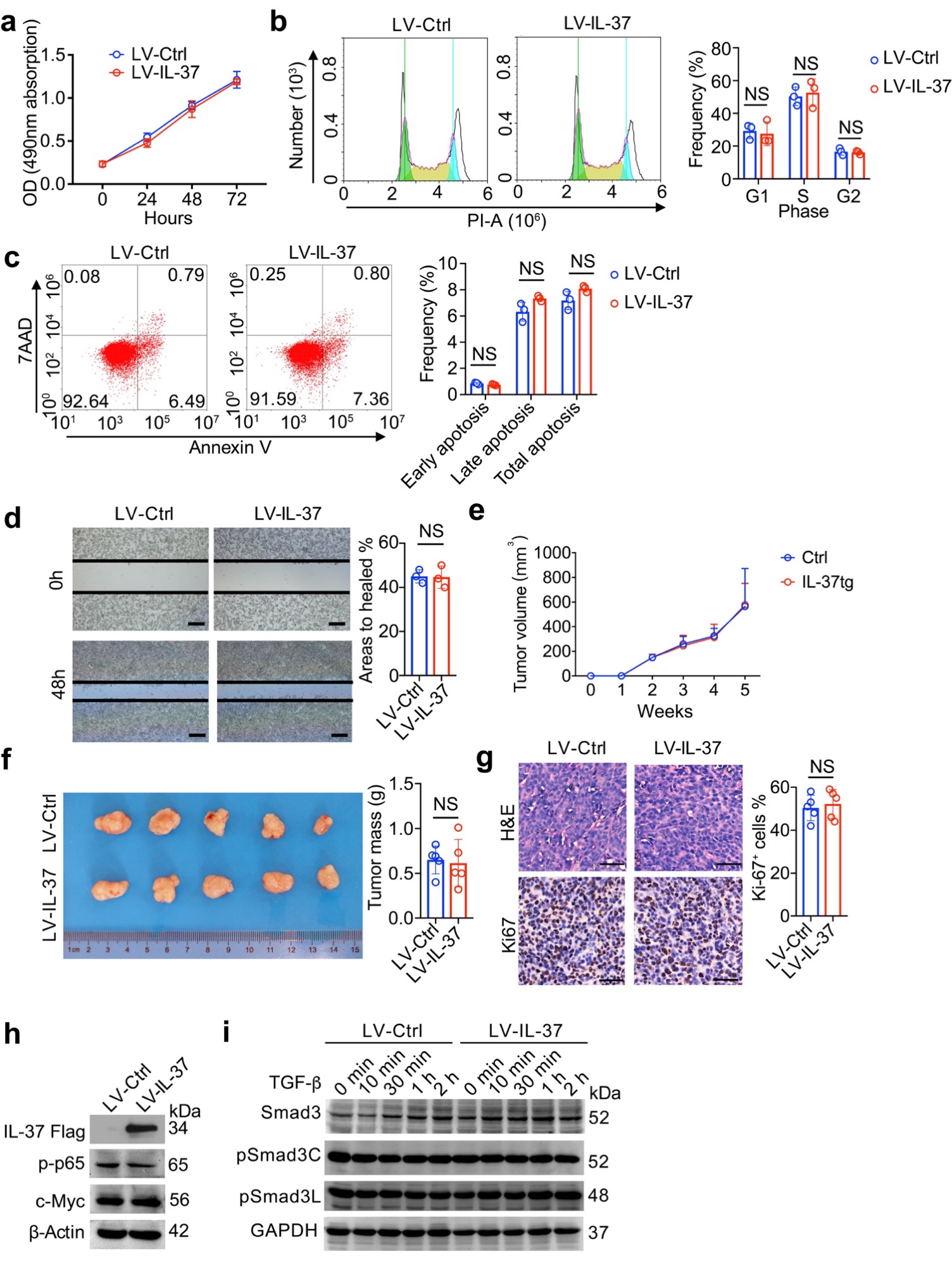


Figure. S4.

**IL-37 did not result in colon tumor cell proliferation. a** Cell growth assays of LV-IL-37 and LV-Ctrl stable LoVo cells were performed using a CCK-8 kit at the indicated times, n=3/group. **b** LV-IL-37 and LV-Ctrl stable LoVo cells were harvested and then stained with propidium iodide (PI), the DNA content was analyzed by flow cytometry. Representative and quantiﬁcation of cell cycle analysis of LV-IL-37 and LV-Ctrl stable LoVo cells. G1, S, and G2 indicate the respective cell cycle phase. n=3/group. **c** LV-Ctrl and LV-IL-37 stable LoVo cells were harvested and stained with Annexin V-PE and 7-AAD. Representative and quantiﬁcation of cell apoptosis analysis of LV-IL-37 and LV-Ctrl stable LoVo cells, n=3/group. **d** LV-Ctrl and LV-IL-37 stable LoVo cells were plated grew to 80–90% confluence and a scratch in the monolayer of cells was generated. Representative photographs of the initial wound area and the same area 48 hours later are provided respectively (left). The width of 5 scratches in 3 independent wells was analyzed for each state. The results represent the areas to be healed (right). Scale bar: 200 μm. **e** Nude mice were grafted with 5 × 10^6^ LV-Ctrl or LV-IL-37 stable LoVo cells at week 0, and the tumor growth was followed until week 5, growth curves of LV-Ctrl and LV-IL-37 stable LoVo colorectal tumor, n=5/group. **f** Representative images of xenograft and tumor volumes were measured at 5 weeks after subcutaneously injected with 5 × 10^6^ LV-Ctrl and LV-IL-37 stable LoVo cells, n=5/group. **g** Representative H&E and immunostaining of Ki-67, and quantitative analysis of Ki-67 positive cells in LV-Ctrl and LV-IL-37 stable LoVo colorectal tumor, scale bar: 50 μm, n=5/group. **h** Representative immunoblots of the indicated proteins from LV-Ctrl and LV-IL-37 stable LoVo cells. β-Actin was used as a loading control. **i** Representative immunoblots of indicated proteins in LV-Ctrl and LV-IL-37 stable LoVo cells stimulated with 20 pM TGF-β in a time-dependent manner. GAPDH was used as a loading control. Data are presented as Mean ± SD. Statistics analyzed by Two-tailed Student’s T-test. NS, not significant.


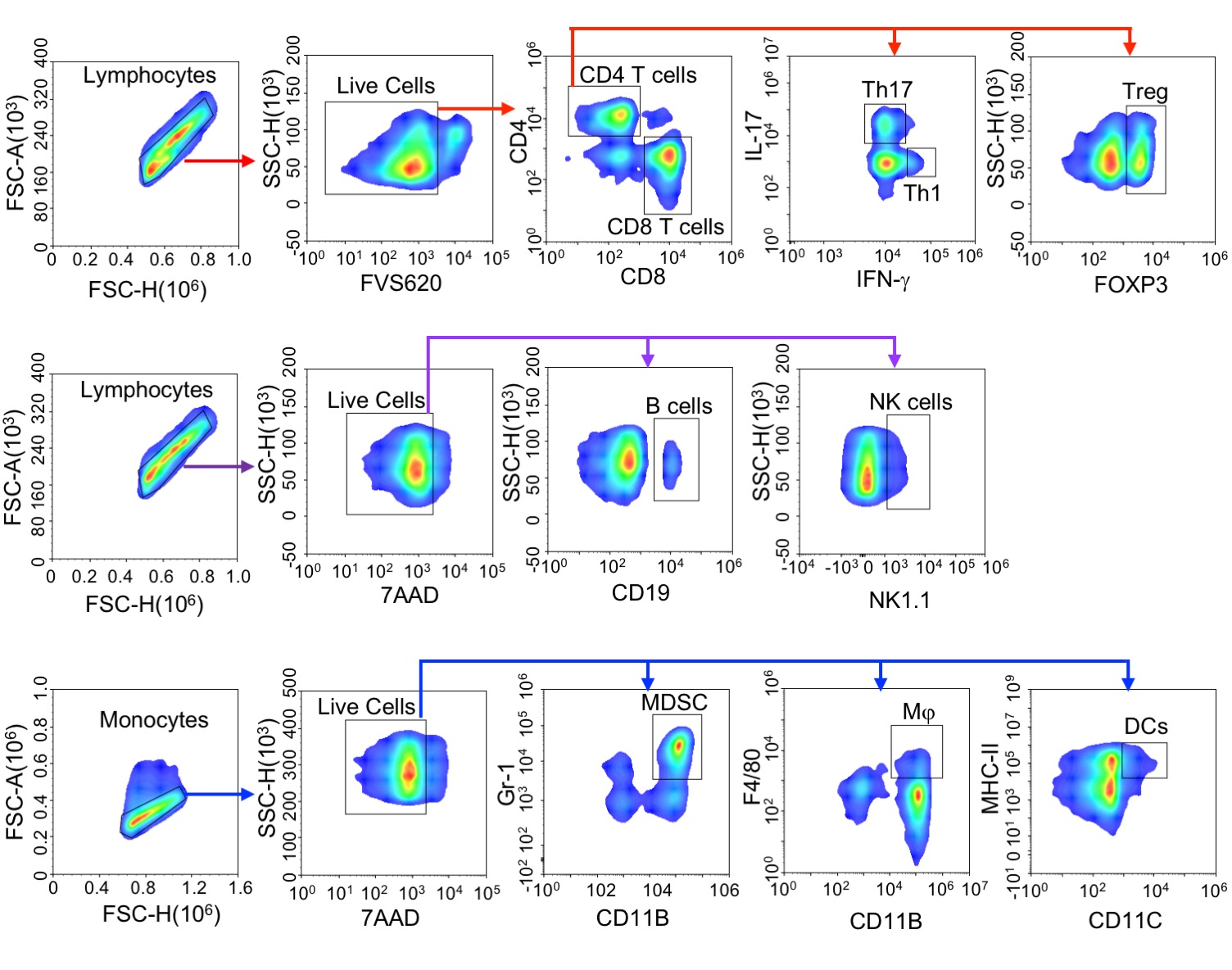


Figure. S5.

**Gating strategy used to identify various immune cells in the tumor-bearing colon tissue by flow cytometry.** Mice were treated with AOM/DSS for 70 days, single-cell suspensions from the tumor-bearing colon tissue were stained with fluorochrome-conjugated antibodies and were analyzed by flow cytometry, lymphocytes and monocytes were eliminated of doublets and debris. Three gating strategies were used to identify populations expressing speciﬁc markers, red line: CD4 cells (CD4^+^), CD8 cells (CD8^+^), Th1 cells (CD4^+^ IFN-γ^+^), Th17 cells (CD4^+^ IL17^+^), Treg (CD4^+^ FOXP3^+^); purple line: B cells (CD19^+^), NK cells (NK1.1^+^); Blue line: MDSC (CD11B^+^Gr-1^+^), Mϕ (CD11B^+^F4/80^+^), DCs (CD11C^+^MHC-II^+^). FSC, forward scatter; MHC II, major histocompatibility complex class II; SSC, side scatter.


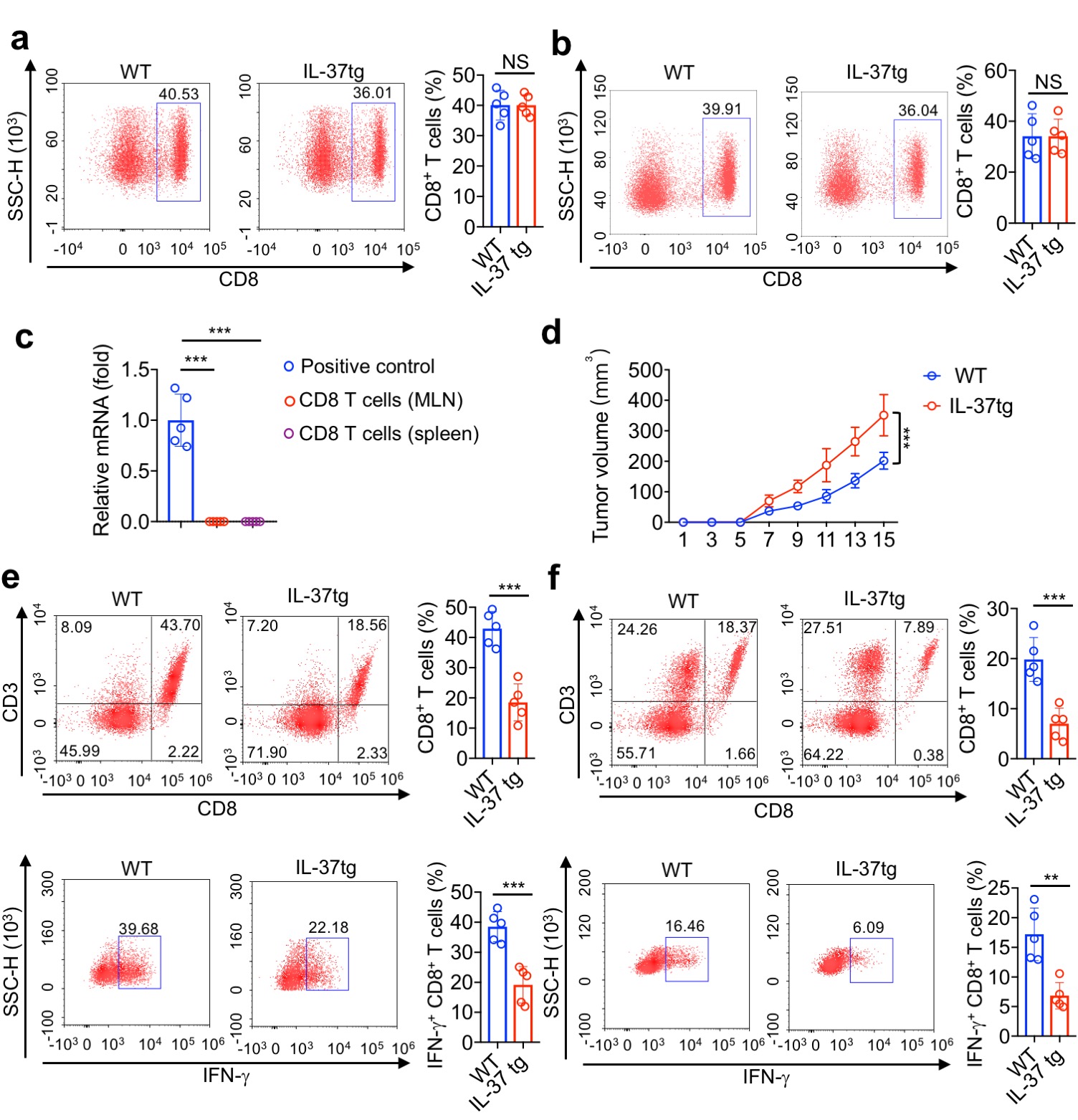
Figure. S6.

**IL-37 damage the effector function of CD8^+^ T cells and enhanced tumor growth in MC38 xenograft model.** **a, b** Representative and quantiﬁcation of FACS analysis of CD8^+^ T cells in the lymph node (**a**) and spleen (**b**) of IL-37tg and WT mice in basal stat. n=5/group. **c** qRT-PCR analysis of the mRNA levels of IL-37 in colon tissues of IL-37tg mice (positive control) and isolated CD8^+^ T cells from the MLN and spleen of IL-37tg mice, n=5/group. **d-f** IL-37tg mice and WT mice were subcutaneous injection of 1 × 10^6^ MC38 cells. **d** Xenograft tumor growth curve of different groups. n=5/group. **e, f** The percentage of CD8^+^ T cells was measured 15 days after tumor inoculation. Representative and quantification of FACS analysis of CD8^+^ T, CD8^+^ T cells expressing activation marker effector molecules IFN-γ in the tumor tissue (**e**) and tumor-draining lymph node (**f**). n=5/group. The data are presented as Mean ± SD. Statistics analyzed by Two-tailed Student’s T-test (**a, b, e, f**), and One-way ANOVA analysis of variance with Turkey’s post hoc test (**c**), Two-way ANOVA analysis of variance with Turkey’s post hoc test (**d**). ***P* < 0.01; ****P* < 0.001. NS, not significant.


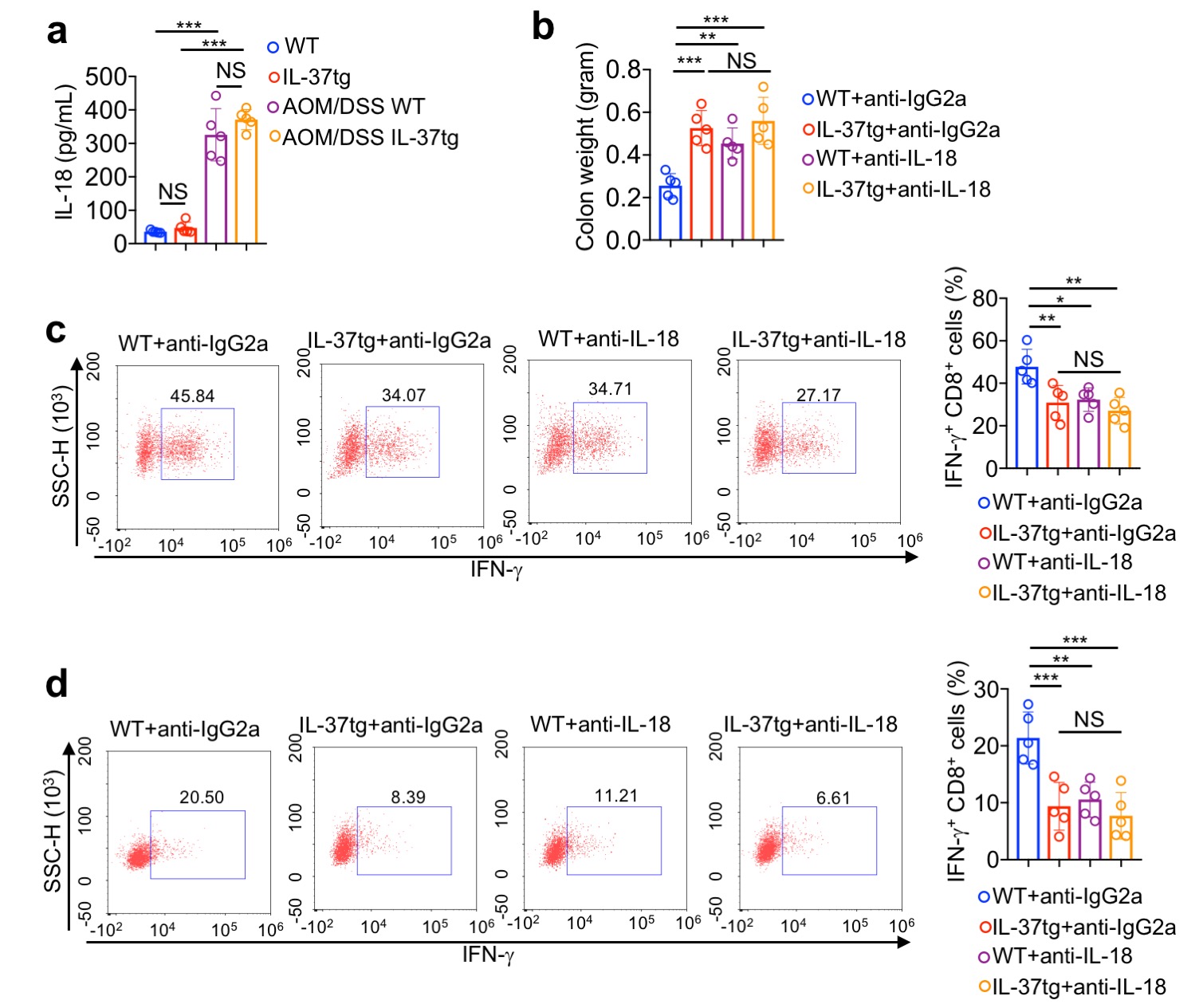


Figure. S7.

**IL-37 inhibited the CD8⁺ cytotoxic T cells–mediated anti-tumor immunity that was depended on IL-18. a-d** IL-37tg mice and WT mice were treated with or without AOM/DSS for 70 days, simultaneously intraperitoneal injection of 50 μg IL-18–neutralizing antibody or IgG2a isotype control twice a week. **a** The colon tissue from IL-37tg mice and WT mice was cut into small pieces and incubated in serum-free RPMI medium for 24 hours. Secreted chemokines and cytokines in the medium were measured by ELISA assay, n=5/group. **b** Colon weight was determined in IL-37tg mice and WT mice, n=5/group. **c-d** Representative and quantification of FACS analysis of CD8^+^ T cells IFN-γ^+^CD8^+^ T cells in the tumor-bearing colon tissue (**c**) and mesenteric lymph nodes (**d**), n=5/group. All data are presented as Mean ± SD. Statistics analyzed by One-way ANOVA analysis of variance with Turkey’s post hoc test. **P* < 0.05; ***P* < 0.01; ****P* < 0.001. NS, not significant.


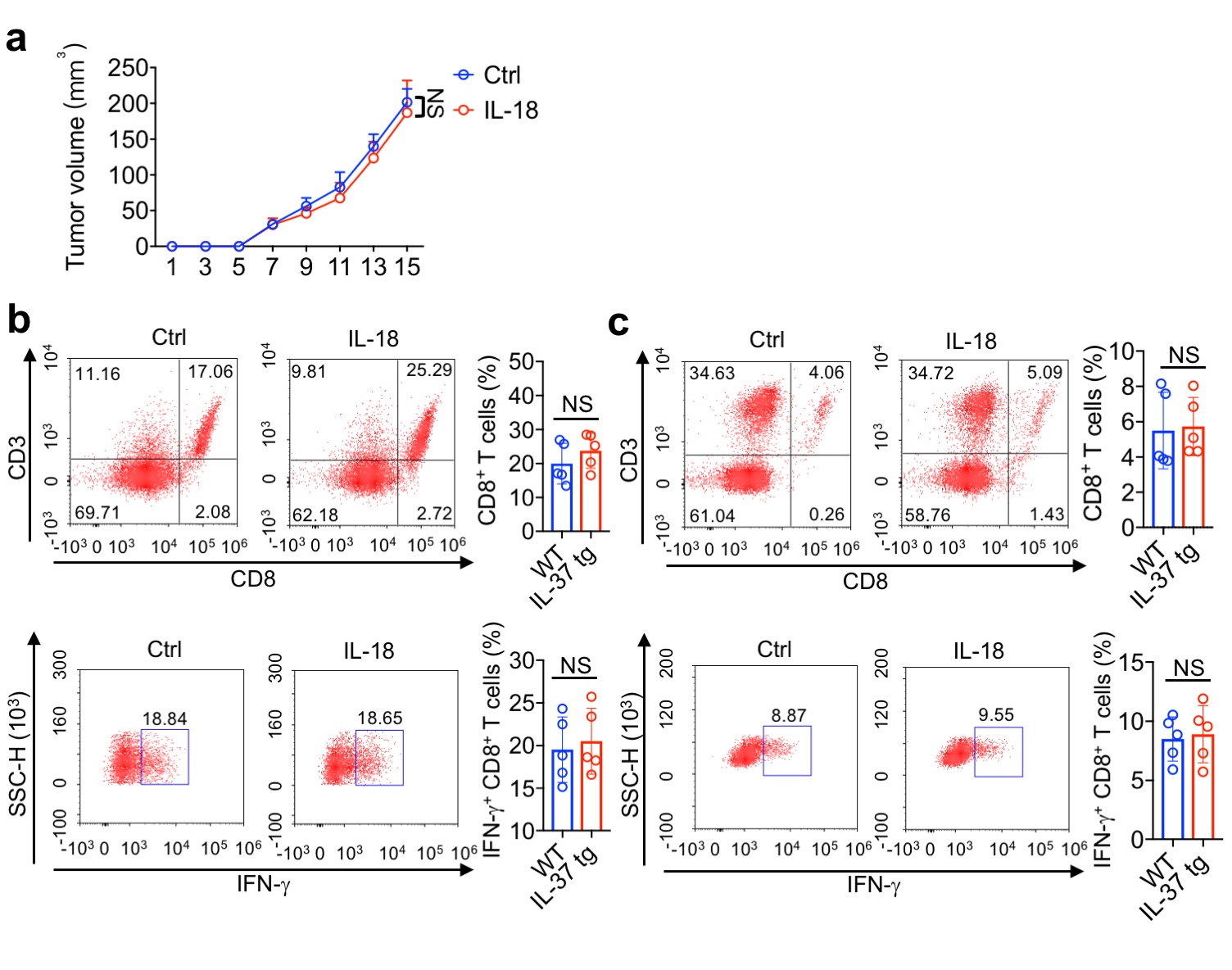


Figure. S8.

**Recombinant IL-18 did not rescue tumor burden and CD8+ T cells inactivation in IL-37tg mice.** **a-c** IL-37tg mice were subcutaneous injection of 1 × 10^6^ MC38 cells, simultaneously intravenous injection of 20 ng recombinant IL-18 or vehicle control every other day. **a** Xenograft tumor growth curve of different groups. **b, c** The percentage of CD8^+^ T cells was measured 15 days after tumor inoculation. Representative and quantification of FACS analysis of CD8^+^ T, CD8^+^ T cells expressing activation marker effector molecules IFN-γ in the tumor tissue (**b**) and tumor-draining lymph node (**c**). n=5/group. The data are presented as Mean ± SD. Statistics analyzed by Two-way ANOVA analysis of variance with Turkey’s post hoc test (**a**) and Two-tailed Student’s T-test (**b, c**). NS, not significant.


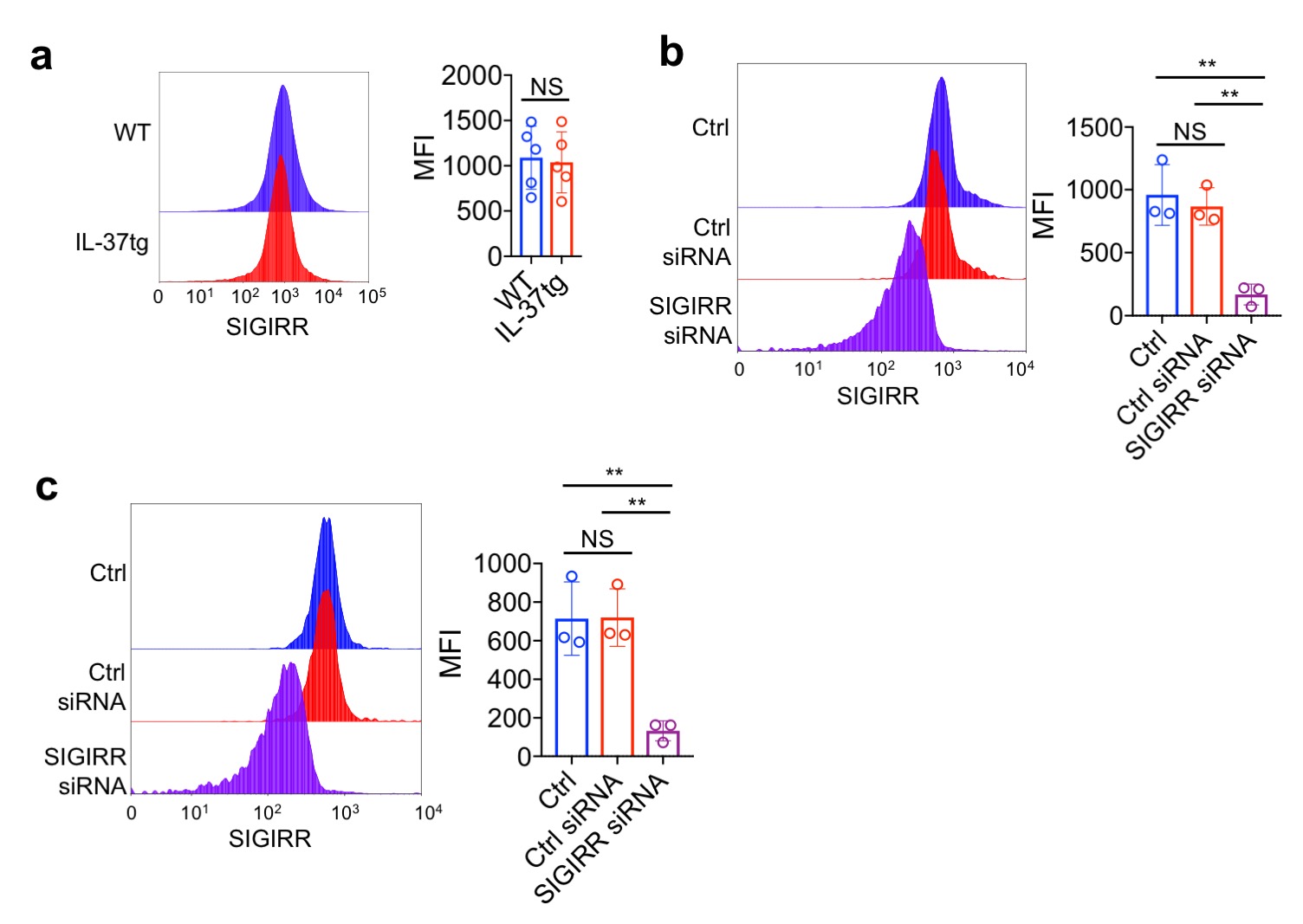


Figure. S9.

**SIGIRR expression and SIGIRR siRNA effectiveness. a** Representative and quantification of FACS analysis of SIGIRR mean fluorescent intensity (MFI) in CD8^+^ T cells from the colon of IL-37tg and WT mice. n=5/group. **b, c** Isolated mouse naive CD8^+^ T cells (**b**) or naive OT-I CD8^+^ T cells (**c**) were transfected for 72 h with the SMARTpool siRNA reagent against SIGIRR or with a control Accell nontargeting siRNA. Representative and quantification of FACS analysis of SIGIRR mean fluorescent intensity (MFI) in transfected cells. n=3/group. The data are presented as Mean ± SD. Statistics analyzed by Two-tailed Student’s T-test (A), and One-way ANOVA analysis of variance with Turkey’s post hoc test (**b, c**). ***P* < 0.01. NS, not significant.


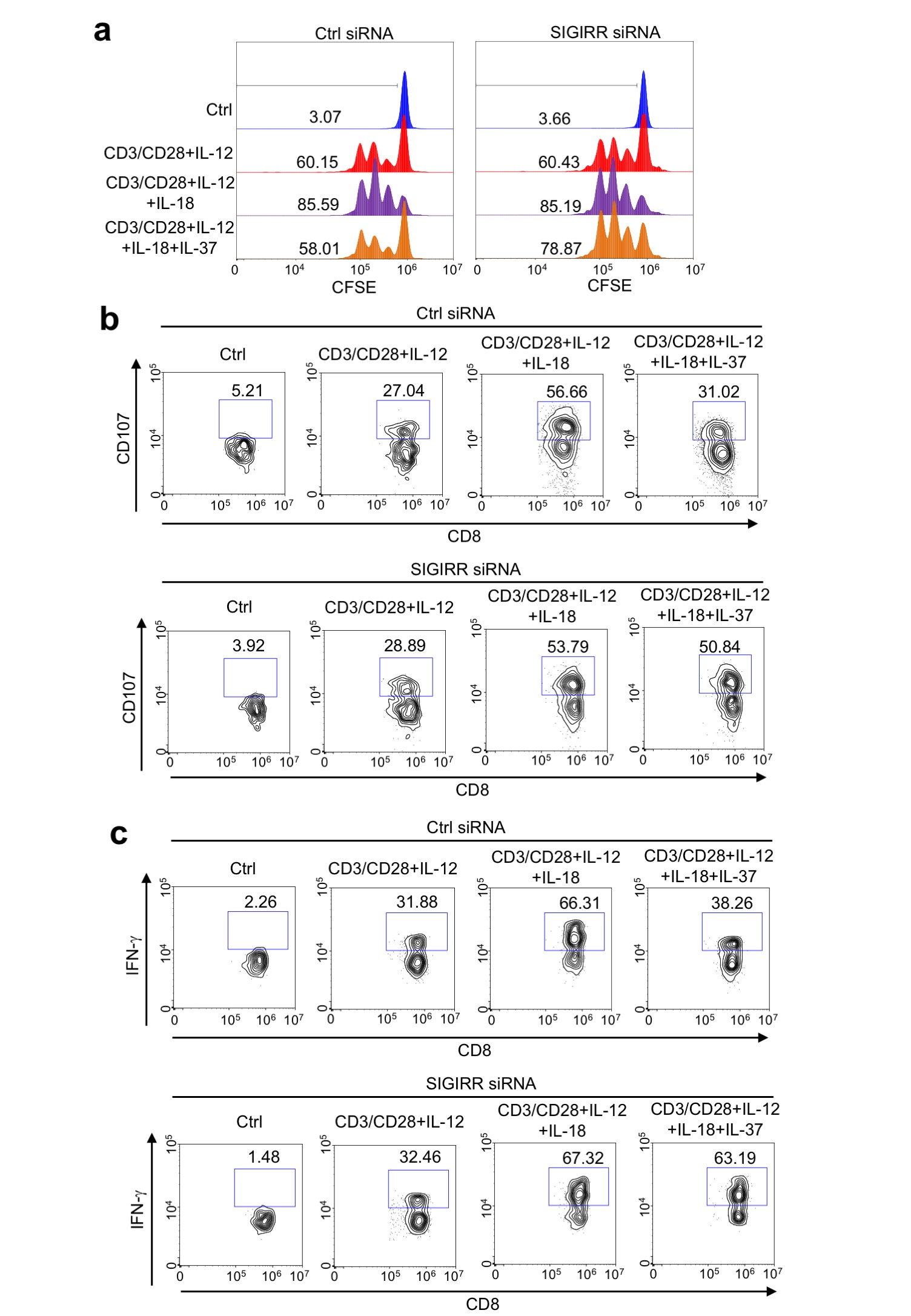


Figure. S10.

**IL-37 limited IL-12/18 induce cytolytic activity of CD8^+^ T depends on SIGIRR upon stimulation with CD3/CD28-coated beads.** **a** Isolated mouse naive CD8^+^ T cells were transfected for 72 h with the SMARTpool siRNA reagent against SIGIRR or with a control Accell nontargeting siRNA, transfected CD8^+^ T cells were labeled with 5 μM CFSE and pulsed with or without CD3/CD28 in the presence or absence of 10 ng/mL IL-12, 100 ng/mL IL-18, 100 ng/mL IL-37b for 72h. Proliferation was determined by the CFSE dilution assay. Representative histograms of CFSE dilution. **b, c** SIGIRR siRNA or Ctrl siRNA transfected CD8^+^ T cells were treated with or without CD3/CD28 in the presence or absence of 10 ng/mL IL-12, 100 ng/mL IL-18, 100 ng/mL IL-37b for 72h. Representative FACS analysis of CD69^+^CD8^+^ cells (**b**) and IFN-γ^+^CD8^+^ cells (**c**).


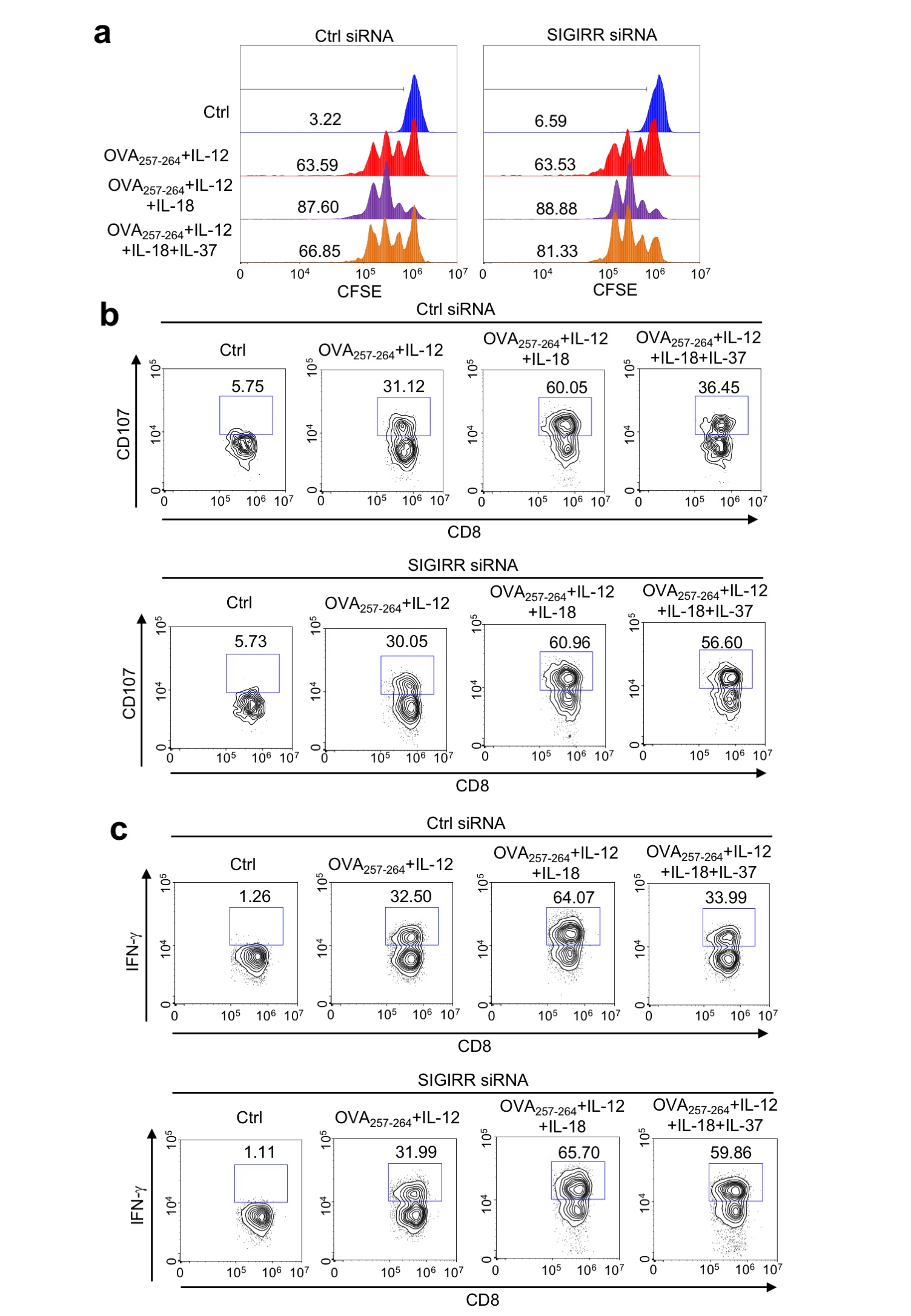
Figure. S11.

**IL-37 limited IL-12/18 induce cytolytic activity of OT-1 CD8+ T cells depends on SIGIRR upon stimulation with OVA257-264. a** Isolated mouse naive CD8^+^ T cells were transfected for 72 h with the SMARTpool siRNA reagent against SIGIRR or with a control Accell nontargeting siRNA, transfected CD8^+^ T cells were labeled with 5 μM CFSE and pulsed with or without 5 μg/mL OVA257-264 peptides in the presence or absence of 10 ng/mL IL-12, 100 ng/mL IL-18, 100 ng/mL IL-37b for 72h. Proliferation was determined by the CFSE dilution assay. Representative histograms of CFSE dilution. **b, c** SIGIRR siRNA or Ctrl siRNA transfected CD8^+^ T cells were treated with or without 5 μg/mL OVA257-264 peptides in the presence or absence of 10 ng/mL IL-12, 100 ng/mL IL-18, 100 ng/mL IL-37b for 72h. Representative FACS analysis of CD69^+^CD8^+^ cells (**b**) and IFN-γ^+^CD8^+^ cells (**c**).

Table S1.

**Characteristics of patients with colorectal cancer.**

| **Case No.** | **Sex** | **Age** | **Tumor status** | | | **Stage** |
| --- | --- | --- | --- | --- | --- | --- |
|  |  |  | **T** | **N** | **M** |  |
| 1 | F | 42 | 3 | 0 | 0 | II |
| 2 | M | 68 | 3 | 0 | 0 | II |
| 3 | M | 51 | 3 | 2 | 0 | III |
| 4 | F | 61 | 3 | 1 | 0 | III |
| 5 | F | 69 | 3 | 1 | 0 | III |
| 6 | M | 55 | 3 | 1 | 0 | III |
| 7 | M | 68 | 4 | 1 | 1 | IV |
| 8 | M | 78 | 3 | 2 | 0 | III |
| 9 | M | 69 | 3 | 1 | 0 | III |
| 10 | M | 35 | 2 | 0 | 0 | I |
| 11 | F | 79 | 3 | 0 | 0 | II |
| 12 | M | 62 | 2 | 0 | 0 | I |
| 13 | M | 59 | 4 | 2 | 1 | IV |
| 14 | M | 62 | 3 | 0 | 0 | II |
| 15 | F | 54 | 3 | 0 | 0 | II |
| 16 | M | 69 | 3 | 2 | 0 | III |
| 17 | F | 69 | 4 | 2 | 1 | IV |
| 18 | F | 59 | 1 | 0 | 0 | I |
| 19 | M | 83 | 3 | 0 | 0 | II |
| 20 | M | 75 | 3 | 0 | 0 | II |
| 21 | F | 58 | 3 | 1 | 0 | III |
| 22 | F | 53 | 3 | 2 | 0 | III |
| 23 | F | 63 | 2 | 0 | 0 | I |
| 24 | M | 31 | 3 | 0 | 0 | II |
| 25 | M | 65 | 3 | 0 | 0 | II |
| 26 | M | 54 | 2 | 0 | 0 | I |
| 27 | F | 53 | 4 | 1 | 0 | III |
| 28 | F | 79 | 3 | 0 | 0 | II |
| 29 | M | 69 | 3 | 0 | 0 | II |
| 30 | F | 63 | 3 | 0 | 0 | II |
| 31 | F | 76 | 4 | 1 | 1 | IV |
| 32 | M | 45 | 3 | 2 | 0 | III |

Table S2.

**Real time qPCR primer sequences.**

| **Gene symbol** | **5' primer** | **3' primer** |
| --- | --- | --- |
| IL-37 | GGACAAAGTCATCCATCCCTTC | GAGCCCACCTGAGCCCTATAA |
| CD24 | GAACCCATCCCCAAGTCCAG | GGTGCTTGTGGTGAGTGAGA |
| c-Kit | AAAGAGCAAATCCAGGCCCA | TAAGGAAGTTGCGTCGGGTC |
| Lgr5 | ATGCGTTTTCTACGTTGCCG | GTCACAGGGAAGGACGACAG |
| Muc1 | GCAGTCCTCAGTGGCACCTC | CACCGTGGGCTACTGGAGAG |
| Muc2 | GCTGACGAGTGGTTGGTGAATG | GATGAGGTGGCAGACAGGAGAC |
| Muc3 | CGTGGTCAACTGCGAGAATGG | CGGCTCTATCTCTACGCTCTCC |
| Muc4 | CAGCAGCCAGTGGGGACAG | CTCAGACACAGCCAGGGAACTC |
| Muc5ac | CTGTGACATTATCCCATAAGCCC | AAGGGGTATAGCTGGCCTGA |
| Tff3 | CCTGGTTGCTGGGTCCTCTG | GCCACGGTTGTTACACTGCTC |
| IL-22 | TTTCCTGACCAAACTCAGCA | CTGGATGTTCTGGTCGTCAC |
| Reg3β | ATGGCTCCTACTGCTATGCC | GTGTCCTCCAGGCCTCTTT |
| Reg3γ | ATGGCTCCTATTGCTATGC | GATGTCCTGAGGGCCTCTT |
| Lcn2 | ACATTTGTTCCAAGCTCCAGGGC | CATGGCGAACTGGTTGTAGTCCG |
| Mptx | CCTGTTTCTCTCTGTTCTTTCAGG | GGCCTTCATACACAGAGTGAAG |
| S100A9 | ACACCTTCCATCAATACTCTA | TCATTTCTCTTCTCTTTCTTCAT |
| Occludin | TTGAAAGTCCACCTCCTTACAGA | CCGGATAAAAAGAGTACGCTGG |
| Zo1 | GCCGCTAAGAGCACAGCAA | GCCCTCCTTTTAACACATCAGA |
| IL-18 | GGCTGCCATGTCAGAAGACT | GTCTGGTCTGGGGTTCACTG |
| IL-4 | ACAGGAGAAGGGACGCCAT | GAAGCCCTACAGACGAGCTCA |
| IFN-γ | TCAAGTGGCATAGATGTGGAAGAA | TGGCTCTGCAGGATTTTCATG |
| IL-6 | GAGAAAAGAGTTGTGCAATGGC | CCAGTTTGGTAGCATCCATCAT |
| TNF-α | GCCACCACGCTCTTCTGTCT | ACTCCAGCTGCTCCTCCACTT |
| Parp1 | ATGGCCAGGACGAAGAGGCAGTAA | TCAGCTTCCCCAAGGGCATCTTCT |
| Msh2 | AGACCCAGGGCGTGATCAAGTACA | TTGCCGGGAGAAGCCTTAAATGCC |
| Msh3 | CAGTTTGTGCCCAACAGTACGAGT | AGCACCCATCCTTGTGAAAATGCC |
| Mlh1 | TCTTCGACTGGCCACTGAGGTGAA | TTTGACGTGGAGCCAGGCATGT |
| Atm | AGCTTGTGAAGGGCCGTGATGA | AACACCGCTTCGCTGAGAAAGG |
| Atr | AGTTGGCCAGTGCTACTCCAGA | GGTCGGCTGAGCGTCAGTTTTCTT |
| Actb | CCTCTATGCCAACACAGTGC | ACATCTGCTGGAAGGTGGAC |
